# Loss of *Vhl* alters trabecular bone loss during *S. aureus* osteomyelitis in a cell-specific manner

**DOI:** 10.3389/fcimb.2022.985467

**Published:** 2022-09-20

**Authors:** Caleb A. Ford, Ian M. Hurford, Laura E. Fulbright, Jacob M. Curry, Christopher T. Peek, Thomas J. Spoonmore, Virginia Cruz Victorio, Joshua R. Johnson, Sun H. Peck, James E. Cassat

**Affiliations:** ^1^ Department of Biomedical Engineering, Vanderbilt University, Nashville, TN, United States; ^2^ Department of Pediatrics, Division of Pediatric Infectious Diseases, Vanderbilt University Medical Center, Nashville, TN, United States; ^3^ Department of Pathology, Microbiology, and Immunology, Vanderbilt University Medical Center, Nashville, TN, United States; ^4^ Department of Chemical and Biomolecular Engineering, Vanderbilt University, Nashville, TN, United States; ^5^ Vanderbilt Center for Bone Biology, Vanderbilt University Medical Center, Nashville, TN, United States; ^6^ Division of Clinical Pharmacology, Department of Medicine, Vanderbilt University Medical Center, Nashville, TN, United States; ^7^ Vanderbilt Institute for Infection, Immunology, and Inflammation (VI4), Vanderbilt University Medical Center, Nashville, TN, United States

**Keywords:** osteomyelitis (OM), *Staphylococcus aureus*, infection, HIF signaling, von Hippel-Lindau (VHL), cre-lox approach, osteoclastogenesis, bone infection

## Abstract

Osteomyelitis, or bone infection, is a major complication of accidental trauma or surgical procedures involving the musculoskeletal system. *Staphylococcus aureus* is the most frequently isolated pathogen in osteomyelitis and triggers significant bone loss. Hypoxia-inducible factor (HIF) signaling has been implicated in antibacterial immune responses as well as bone development and repair. In this study, the impact of bone cell HIF signaling on antibacterial responses and pathologic changes in bone architecture was explored using genetic models with knockout of either *Hif1a* or a negative regulator of HIF-1α, *Vhl*. Deletion of *Hif1a* in osteoblast-lineage cells *via Osx-Cre* (*Hif1a^ΔOB^*) had no impact on bacterial clearance or pathologic changes in bone architecture in a model of post-traumatic osteomyelitis. Knockout of *Vhl* in osteoblast-lineage cells *via Osx-Cre* (*Vhl^ΔOB^*) caused expected increases in trabecular bone volume per total volume (BV/TV) at baseline and, intriguingly, did not exhibit an infection-mediated decline in trabecular BV/TV, unlike control mice. Despite this phenotype, bacterial burdens were not affected by loss of *Vhl*. *In vitro* studies demonstrated that transcriptional regulation of the osteoclastogenic cytokine receptor activator of NF-κB ligand (RANKL) and its inhibitor osteoprotegerin (OPG) is altered in osteoblast-lineage cells with knockout of *Vhl*. After observing no impact on bacterial clearance with osteoblast-lineage conditional knockouts, a *LysM-Cre* model was used to generate *Hif1a^ΔMyeloid^* and *Vhl^ΔMyeloid^* mouse models to explore the impact of myeloid cell HIF signaling. In both *Hif1a^ΔMyeloid^* and *Vhl^ΔMyeloid^* models, bacterial clearance was not impacted. Moreover, minimal impacts on bone architecture were observed. Thus, skeletal HIF signaling was not found to impact bacterial clearance in our mouse model of post-traumatic osteomyelitis, but *Vhl* deletion in the osteoblast lineage was found to limit infection-mediated trabecular bone loss, possibly *via* altered regulation of RANKL-OPG gene transcription.

## Introduction

Osteomyelitis is a highly morbid bone disease that is most commonly caused by infection with *Staphylococcus aureus*, a Gram-positive opportunistic pathogen that colonizes about one in three individuals (Lew and Waldvogel, 2004; Wertheim et al., 2005; Kremers et al., 2015). Osteomyelitis is difficult to treat because bacterial pathogens can often evade antibacterial immune responses and tolerate antimicrobial therapy (Ford and Cassat, 2017; Muthukrishnan et al., 2019). Bone infection frequently results in chronic disease and concomitant bone destruction (Lew and Waldvogel, 2004). Therefore, curative treatment of osteomyelitis requires both bacterial clearance and repair of pathologic bone loss.

Hypoxia-inducible factor (HIF) signaling is a critical cell signaling pathway in response to *S. aureus* skin infection and in fracture healing (Wan et al., 2008; Wickersham et al., 2017). HIF signaling is activated in response to hypoxia, a hallmark of many infectious niches, including infected bone (Schaffer and Taylor, 2015; Wilde et al., 2015; Devraj et al., 2017). *S. aureus* modulates its virulence in response to tissue hypoxia during osteomyelitis, and bacterial responses to hypoxia are critical for staphylococcal survival in bone (Wilde et al., 2015). However, the impact of host hypoxic responses during *S. aureus* osteomyelitis is unknown. In normoxia, HIF-1α activity is inhibited by the hydroxylation of proline residues within the oxygen-dependent domain of the HIF-1α subunit, which is accomplished by prolyl hydroxylase domain (PHD) proteins (Huang et al., 1998; Ivan et al., 2001; Jaakkola et al., 2001). Following hydroxylation, von Hippel Lindau protein (VHL) polyubiquitinates HIF-1α, targeting it for rapid proteasomal degradation (Wang et al., 1995; Salceda and Caro, 1997; Maxwell et al., 1999). Canonically, the HIF-1α subunit is stabilized in hypoxic environments through inhibition of PHD hydroxylation (Semenza and Wang, 1992; Kaelin and Ratcliffe, 2008). Stabilized HIF-1α heterodimerizes with HIF-1β (aryl hydrocarbon receptor nuclear translocator [ARNT]) and binds with a coactivator CREB-binding protein (CBP) or p300 (Wang et al., 1995; Arany et al., 1996; Ema et al., 1999). The active HIF transcriptional complex regulates a wide range of genes containing hypoxia response elements (HREs), which are principally genes of metabolic and angiogenic pathways (Kaelin and Ratcliffe, 2008).

HIF signaling has the potential to ameliorate bone damage and bacterial burdens during osteomyelitis based on prior investigations in bone biology and, separately, in host-pathogen interactions. Genetic or pharmacologic augmentation of HIF signaling enhances bone formation in response to sterile injury (Wan et al., 2008; Shen et al., 2009; Woo et al., 2015; Deng et al., 2019). Activation of HIF signaling in osteoblasts improves bone formation in part by shifting the metabolism of osteoblasts toward glycolysis and increasing the production of vascular endothelial growth factor (VEGF) (Liu et al., 2012; Regan et al., 2014; Hu and Olsen, 2016). Moreover, activation of HIF signaling in osteoblast-lineage cells has been shown to negatively regulate the formation of bone-resorbing osteoclasts (Wu et al., 2015). HIF-1α is also essential for myeloid inflammatory responses (Cramer et al., 2003). HIF-1α prolongs myeloid cell longevity in the infectious niche and increases production of antimicrobial peptides, cytokines, and reactive oxygen species. Moreover, shifting of the cell metabolism toward a glycolytic state by HIF-1α supports the energetic demands of activated myeloid cells in other models of inflammation (Cramer et al., 2003; Walmsley et al., 2005; Nizet and Johnson, 2009). Furthermore, HIF signaling enhances antibacterial responses in non-professional immune cells such as keratinocytes during *S. aureus* skin infection (Wickersham et al., 2017). It is therefore possible that HIF signaling has a similar function in bone cells.

As a critical signaling pathway during bone repair and antibacterial immune responses, HIF signaling may represent a tractable pharmacologic target for adjunctive osteomyelitis therapy. In this study, we investigated the impact of HIF signaling in cells of the osteoblast and myeloid lineages. To accomplish this, we used two different Cre recombinase models to conditionally delete *Vhl* or *Hif1a* to model states of high and low HIF signaling. Using these genetic models, we studied the role of HIF signaling on bacterial clearance and pathologic changes to bone architecture in a mouse model of *S. aureus* osteomyelitis.

## Materials and methods

### Bacterial strains and reagents

An erythromycin-sensitive derivative of USA300 LAC *S. aureus* (AH1263) was used for *in vivo* infections and is the parent strain of the toxin-deficient strain used *in vitro* (Boles et al., 2010). The toxin-deficient strain used *in vitro* was generated by inactivating *spa* in LAC Δ*psmα1-4*::*erm* (Δ*psm*), a mutant which has been characterized and used previously (Cassat et al., 2013; Putnam et al., 2019). To generate a double knockout of *psmα1–4* and *spa*, Δ*spa*::Tc^r^ was transduced into Δ*psm* from Newman Δ*spa*::Tc^r^ (DU5873) with the bacteriophage phi-85 as previously described (Hartleib et al., 2000; Wilde et al., 2015). The double knockout strain (herein referred to as Δ*psm*/Δ*spa*) limits cell death in *in vitro* assays and limits potential interference of staphylococcal protein A in downstream, antibody-based assays. The toxin-deficient strain used in all *in vitro* experiments refers to the Δ*psm*/Δ*spa* strain. Bacterial growth media and other reagents used are products of MilliporeSigma (Burlington, MA, USA) unless otherwise stated. Bacteria were grown on tryptic soy agar (TSA) or in a shaking incubator at 37°C in tryptic soy broth (TSB). Tetracycline (2 μg/mL) was added for growth of DU5873. Erythromycin (10 μg/mL) was added for growth of the Δ*psm* and Δ*psm*/Δ*spa*. Bacteria (Δ*psm*/Δ*spa*) were grown in RPMI (Corning, Corning, NY, USA) with 10 g/L casamino acids for growth prior to harvesting and concentration of bacterial supernatants as done previously (Wilde et al., 2015). To prepare the bacterial inoculum for *in vivo* infection, bacteria were grown in TSB overnight at 37°C on an orbital shaker. Then, the overnight bacterial culture was sub-cultured in TSB at a 1:100 dilution for 3 h before centrifuging to pellet the cells and resuspending in phosphate-buffered saline (PBS, Corning) as described previously (Klopfenstein et al., 2021).

### Mouse strains

*OsxCre* (Jackson Labs #006361), *Vhl^fl/fl^* (Jackson Labs #012933), and *Hif1a^fl/fl^* (Jackson Labs #007561) mice were purchased from Jackson Labs (Bar Harbor, ME, USA) (Ryan et al., 2000; Haase et al., 2001; Rodda and McMahon, 2006). *LysMCre^+/+^* (Jackson Labs #004781) mice were obtained from Dr. Carlos H. Serezani (Clausen et al., 1999). Cre mice and mice containing floxed alleles were crossed in combination to generate conditional knockout mice: *Vhl^fl/fl^*, *OsxCre+* (*Vhl^ΔOB^*); *Hif1a^fl/f^*, *OsxCre+* (*Hif1a^ΔOB^*); *Vhl^fl/fl^*, *LysMCre^+/-^* (*Vhl^ΔMyeloid^*); and *Hif1a^fl/f^*, *LysMCre^+/-^* (*Hif1a^ΔMyeloid^*). Thus, the *LysMCre* conditional knockout mice contained one wildtype *Lyz2* allele and one nonfunctional *Lyz2* allele due to the insertion of *cre* into the transgenic copy of *Lyz2*. The *OsxCre* mice were maintained with only one copy of the bacterial artificial chromosome with the *cre*-containing allele. Cre-negative littermate controls consisted of mice with corresponding homozygous or heterozygous floxed alleles, as shown in the figure legends, and no copy of *cre*. Some experiments, specified in figure legends, use a control set from a combination of homozygous and heterozygous floxed mice (fl/fl and fl/+). In these experiments, the homozygous and heterozygous control combination uniformly lacked *cre* and showed no phenotypic differences (data not shown). In some studies, age-matched, Cre-positive controls were used, as noted in figure legends, that were either *OsxCre+* (hemizygous) or *LysMCre^+/-^* (heterozygous). Mice bred in the *OsxCre*-containing colonies were maintained on 2 mg/mL doxycycline water to suppress Cre activity. For experiments, mice in the *OsxCre*-containing colonies had doxycycline withdrawn at postnatal age 4 weeks except when noted otherwise. The progeny used in experiments were ear-punched and genotyped by Transnetyx, Inc. (Cordova, TN, USA), using RT-PCR.

### Murine model of osteomyelitis

All experiments involving animals were reviewed and approved by the Institutional Animal Care and Use Committee at Vanderbilt University Medical Center on protocols M12059 and M1800055. All experiments were performed according to NIH guidelines, the Animal Welfare Act, and US federal law. Osteomyelitis was induced in 7- to 8-week-old mice as described previously (Cassat et al., 2013). Anesthesia was maintained with isoflurane (1-5%). Briefly, the anterolateral surface of the femur was exposed, and an approximately 1-mm unicortical bone defect was created by trephination of bone near the mid-diaphysis to access the medullary space. After accessing the medullary space, 10^6^ colony forming units (CFU) of *S. aureus* in 2 μL PBS were directly inoculated into the intramedullary space. In a subset of experiments, 10^5^ CFU were inoculated, as noted. Peri-operative analgesia was provided pre-operatively and every 8–12 h for 48 h post-infection [buprenorphine 0.05–0.1 mg/kg (Reckitt Benckiser, Slough, England, UK)] or pre-operatively as a slow-release formula [buprenorphine-SR 1 mg/kg (ZooPharm, Laramie, WY, USA)] as described previously (Putnam et al., 2019; Ford et al., 2021). For the first 72 h post-infection, mice were monitored and weighed daily. Following 72 h post-infection, mice were weighed and monitored at twice weekly intervals, at a minimum. The infection was continued for up to 14 days. In consultation with the veterinary staff, mice that experienced greater than 20% weight loss were euthanized as a humane endpoint. These mice were excluded from studies. Mice were euthanized by CO_2_ asphyxiation with cervical dislocation as secondary confirmation.

### Quantification of bacterial burdens

At post-infection days 3, 5, or 14, mice were euthanized, and bacterial burdens were analyzed as reported previously (Putnam et al., 2019). The femurs were dissected using sterile technique and homogenized in 500 μL CelLytic Buffer MT Cell Lysis Reagent (MilliporeSigma) using a BulletBlender and NAVY lysis tubes (Next Advance, Inc., Troy, NY, USA). The homogenates were serially diluted in PBS and plated for CFU enumeration on TSA plates.

### Quantification of cytokine abundances in femurs

Following homogenization as performed for quantification of bacterial burdens, homogenates of infected and contralateral femurs were analyzed using a Millipore Luminex multiplexed cytokine kit (MCYTOMAG-70K, MilliporeSigma) according to manufacturer’s instructions. Cytokines below the limit of detection for all experimental conditions or with insufficient bead counts were excluded from analysis. Bulk protein was used to normalize cytokine concentration measurements using Pierce BCA Protein Assay Kit (ThermoFisher Scientific, Waltham, MA) following manufacturer instructions.

### Analysis of bone architecture by microcomputed tomography

Microcomputed tomography (microCT) was used to measure changes in cortical and trabecular bone as previously reported (Cassat et al., 2013; Putnam et al., 2019). Following euthanasia at post-infection day 14, dissected femurs were scanned using a microCT 50 (Scanco Medical, Switzerland) at 10-μm voxel size at 70 kV, 200 μA, and an integration time of 350 ms in a 10.24-mm view, resulting in 1088 slices of the femur that included the diaphysis and the distal metaphysis. Scans were analyzed using microCT V6.3–4 software (Scanco USA, Inc., Wayne, PA, USA).

Cortical bone destruction was measured by drawing contours around the endosteal and periosteal surfaces for 818 slices surrounding the cortical defect as done previously (Putnam et al., 2019). In *Vhl^ΔOB^* (*Vhl^fl/fl^*, *OsxCre+*) mice and their Cre-negative littermate controls, cortical bone loss was measured by drawing contours along the periosteal surface for 409 slices surrounding the cortical defect in the infected femur and 409 slides surrounding the corresponding mid-diaphysis in the contralateral femurs. For *Vhl^ΔOB^* mice and their Cre-negative littermate controls, cortical bone loss is presented as normalized cortical bone loss. Normalized cortical bone loss was calculated by dividing the quantified cortical bone loss per total contoured volume in the infected femur by the same calculated value in the contralateral femur of the given animal. This alternative approach was used to limit the confounding influence of trabecularization of cortical bone in the *Vhl^ΔOB^* mice, as has been noted previously (Dirckx et al., 2018). This normalization also accounts for artifacts from increased vascularity in the cortical bone of *Vhl^ΔOB^* mice that may be falsely measured as infection-mediated bone loss. Trabecular bone volume per total volume (BV/TV) was measured as done previously (Putnam et al., 2019). Briefly, trabecular bone (excluding cortical bone) in the distal metaphysis of the femur was analyzed across 101 slices 300 μm (30 slices) from the growth plate to measure BV/TV.

### Bone histomorphometry

Following microCT scanning, femurs were analyzed for osteoclast abundance in the distal metaphyseal trabecular bone using histomorphometry, as done previously (Putnam et al., 2019). Femurs were decalcified in 20% EDTA for 3 days, paraffin embedded, and sectioned on a Leica RM2255 microtome (Leica Biosystems, Buffalo Grove, IL, USA) at a thickness of 4 μm. Sections were stained for tartrate-resistant acid phosphatase (TRAP) with hematoxylin counterstain. TRAP-stained slides were imaged with a Leica SCN400 Slide Scanner (Leica Biosystems) in brightfield at 20X or on a Cytation 5 Imaging System [BioTek Instruments, Winooski, VT, USA] in brightfield at 10X and analyzed with Bioquant software (Bioquant Image Analysis Corporation, Nashville, TN, USA). Bioquant software was used to measure osteoclast number, osteoclast surface, and bone surface in the trabeculae of the distal metaphysis of the femur proximal to the growth plate on the TRAP-stained slides. According to ASBMR standards (Dempster et al., 2013), osteoclast number, osteoclast surface, and bone surface were measured to calculate osteoclast number per bone surface (N.Oc/BS) and osteoclast surface per bone surface (Oc.S/BS).

### Concentration of bacterial supernatants

Bacterial supernatants for Δ*psm*/Δ*spa* were prepared as done previously (Wilde et al., 2015). In brief, supernatants from overnight cultures of bacteria grown in RPMI supplemented with 10 g/L casamino acids were centrifuged in Amicon^®^ Ultra 3 kDa molecular weight cutoff filter tubes (MilliporeSigma) to concentrate 45 mL to approximately 1.5 mL. Filter-sterilized, concentrated supernatants were aliquoted and stored at -80°C until thawed for use in *in vitro* experiments.

### Primary osteoblast isolation

Primary calvarial osteoblasts were obtained from neonatal *Vhl^fl/fl^* mice as described previously (Johnson et al., 2014). Briefly, calvariae were dissected from 24- to 48-h-old mice and digested five times in dispase (MilliporeSigma) and type II collagenase (MilliporeSigma). Digest fractions 2-5 were pooled and plated on tissue-culture treated 10-cm dishes (Corning) in α-minimal essential media (α-MEM; Gibco #A1049001; ThermoFisher Scientific) containing 10% fetal bovine serum (FBS; Bio‐Techne) and 1x penicillin/streptomycin (ThermoFisher Scientific). At near confluency, the cells were passaged in 0.25% trypsin with 2.21 mM EDTA (Corning) to 24-well plates for experiments and maintained in α-MEM with 10% FBS and 1x penicillin/streptomycin.

### Adenovirus infection and bacterial stimulation

Primary calvarial osteoblasts were treated with adenovirus (Vector Biolabs, Malvern, PA, USA) containing green fluorescent protein (GFP) or adenovirus containing Cre recombinase and GFP (AdGFP or AdCre, respectively) at a multiplicity of infection of 100. Three days following infection with adenovirus, the media was removed and replaced with fresh media containing 5% (v/v) bacterial supernatants from Δ*psm*/Δ*spa S. aureus*. The vehicle control for bacterial supernatants was RPMI media (Corning) containing 10 g/L casamino acids (MilliporeSigma). 6 h following bacterial supernatant exposure, cells were washed in PBS and lysed in RLT buffer (Qiagen Sciences, Germantown, MD, USA) containing 1% (v/v) β-mercaptoethanol (MilliporeSigma).

### RT-PCR

RNA was isolated from primary osteoblasts using the RNeasy Mini Kit (Qiagen Sciences) according to manufacturer’s instructions with on-column treatment with RNase-Free DNase (Qiagen Sciences). Following isolation, cDNA was synthesized using qScript™ cDNA SuperMix (QuantaBio, Beverly, MA, USA) according to manufacturer’s instructions. RT-PCR was conducted in a thermocycler using diluted cDNA, with primers as shown (**Table 1**), and iQ SYBR Green Supermix (Bio-Rad, Hercules, CA, USA) for up to 40 cycles with a melting temperature of 55°C. Fold change in gene expression was determined by normalizing the threshold values (*C*_T_) by the vehicle (RPMI)-AdGFP condition according to the 2^-ΔΔ^ *^C^* ^T^ method as described previously (Schmittgen and Livak, 2008).

**Table 1:** Primer sequences used for RT-PCR.

Gene		Sequences	PrimerBank ID
*Actb* (β-actin)	Forward	5’-GGCTGTATTCCCCTCCATCG-3’	6671509a1
	Reverse	5’-CCAGTTGGTAACAATGCCATGT-3’	
*Tnfsf11* (RANKL)	Forward	5’-CAGCATCGCTCTGTTCCTGTA-3’	6755833a1
	Reverse	5’-CTGCGTTTTCATGGAGTCTCA-3’	
*Tnfrsf11b* (OPG)	Forward	5’-ACCCAGAAACTGGTCATCAGC-3’	31543882a1
	Reverse	5’-CTGCAATACACACACTCATCACT-3’	
*Vhl* (VHL)	Forward	5’-ACATCGTCAGGTCACTCTATGA-3’	118130344c3
	Reverse	5’-CTCTTGGCTCAGTCGCTGTAT-3’	
*Sp7* (Osx)	Forward	5’-ATGGCGTCCTCTCTGCTTG-3’	18485518a1
	Reverse	5’-TGAAAGGTCAGCGTATGGCTT-3’	

For each gene (protein in parentheses) tested by RT-PCR, the forward and reverse primer sequences are shown along with the Harvard PrimerBank ID for each primer pair. PrimerBank (available at: https://pga.mgh.harvard.edu/primerbank/index.html) was used as the reference for each primer set.

In a separate experiment, whole bone marrow was isolated from *Vhl^ΔMyeloid^* mice and their controls (age-matched *LysMCre^+/-^* mice and littermate *Vhl^fl/fl^* mice) and differentiated into bone marrow-derived macrophages using methods previously published (Putnam et al., 2019). Using identical methods for RNA isolation from the primary osteoblasts, RNA from the differentiated bone marrow-derived macrophages was isolated, and RT-PCR was performed using the primers for *Vhl* and *Actb* (**Table 1**) and quantified using the 2^-ΔΔ^ *^C^* ^T^ method.

### Statistical analysis

**Figure 1:**
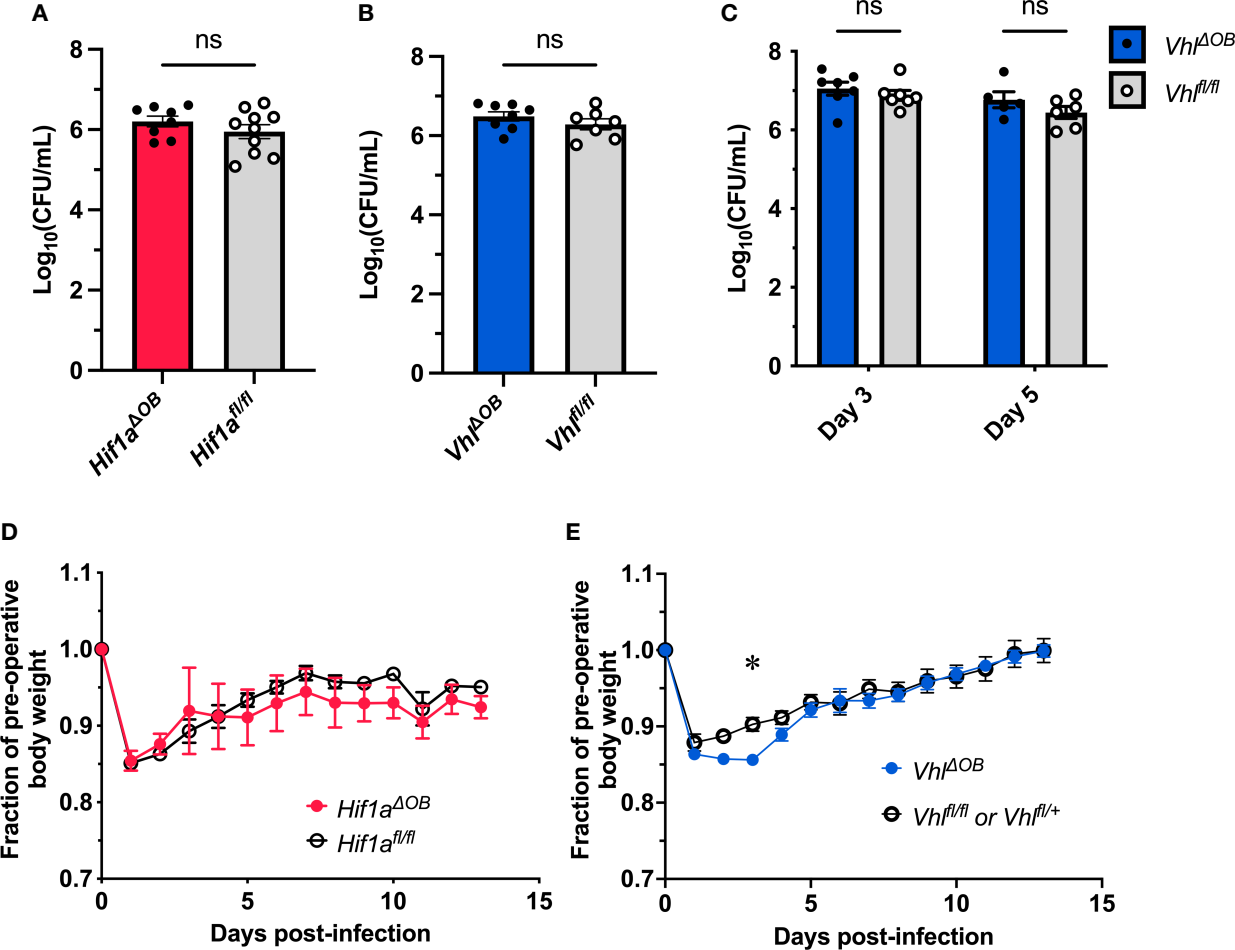
Conditional knockout of *Hif1a* or *Vhl* in the osteoblast lineage does not impact bacterial burdens. Bacterial burdens in infected femurs of female *Hif1a^ΔOB^* **(A)** and *Vhl^ΔOB^* **(B)** mice were measured at post-infection day 14 (*n*=7-10, as shown). The bacterial burdens were compared to those of Cre-negative littermate controls that were homozygous for the respective floxed alleles. ns denotes not significant (*p* > 0.05) as determined by unpaired, two-tailed Student’s *t*-tests. **(C)** Bacterial burdens of *Vhl^ΔOB^* mice were measured at post-infection day 3 and day 5. The bacterial burdens of conditional knockout mice were compared to those of *Vhl^fl/fl^* littermate controls (*n*=5-7 female mice as shown). The relative weights of the corresponding infected animals analyzed in **(A, B)** are shown in **(D, E)**, respectively. The weights were measured daily and normalized to the pre-operative, uninfected animal weights before comparison by two-way ANOVA with multiple comparisons. The weights of *Hif1a^ΔOB^* mice **(D)** were not found to significantly differ (not shown) from those of controls. Error bars represent mean ± SEM. *p < 0.05 as determined by two‐way analysis of variance (ANOVA) with multiple comparisons **(C-E)**. CFU, colony forming units.

Differences in post-infection weights, trabecular bone microCT analysis, histomorphometry measurement of osteoclasts *in vivo*, and RT-PCR following AdCre treatment were assessed by two‐way analysis of variance (ANOVA) with correction for multiple comparisons as noted in the figure legends. The analyzed variables for each two-way ANOVA are defined in figure legends. Differences in trabecular bone microCT analysis in **Figure 3C** were assessed by two-tailed, paired *t*-test. Differences in bacterial burdens were generally assessed by two-tailed, unpaired Student’s *t*‐test. Bacterial burdens in **Figure 5C** were compared by one-way ANOVA, with the analyzed variable being bacterial burdens as Log_10_(CFU/mL). Bacterial burdens in **Figure 1C** were compared by two-way ANOVA with multiple comparisons. Differences in cortical bone loss and *Vhl* transcription in bone marrow-derived macrophages were assessed by two-tailed, unpaired Student’s *t*‐test. A *p* value of 0.05 was considered significant for all analyses. All statistical analyses were performed with GraphPad Prism Version 9.3.1 (GraphPad Software, LLC, San Diego, CA, USA).

## Results

### Conditional knockout of *Vhl* or *Hif1a* in osteoblast-lineage cells does not alter bacterial burdens at post-infection day 14

Osteoblast-lineage conditional deletion of *Vhl* or *Hif1a* was achieved through Cre-lox breeding employing *OsxCre* mice and *Vhl^fl/fl^* or *Hif1a^fl/fl^* mice, respectively. The *OsxCre* model employs a transgene that enables suppression of Cre-mediated conditional deletion of floxed alleles through a Tet-off system (Rodda and McMahon, 2006). With this transgene, doxycycline in the water suppresses Cre recombinase expression and activity to delay conditional gene deletion. Prior studies have demonstrated that constitutively active deletion of *Vhl* in osteoblasts causes profound changes in bone architecture (Wang et al., 2007; Dirckx et al., 2018). To limit baseline differences in bone architecture that may impact bacterial clearance, doxycycline was administered to delay onset of Cre-mediated gene deletion. Based on previously published studies using the *OsxCre* mouse line (Hu and Olsen, 2016), Cre activity was suppressed with continuous doxycycline exposure during *in utero* and early postnatal development until postnatal age 4 weeks, when doxycycline was withdrawn. Following 3 to 4 weeks of doxycycline washout and Cre activation, the infection took place at postnatal age 7 to 8 weeks of age.

Bacterial burdens recovered from infected femurs of female *Hif1a^ΔOB^* or *Vhl^ΔOB^* mice did not differ from their respective littermate controls that lack the *OsxCre* transgene (**Figures 1A–C**). Over the course of infection, the relative weight loss did not significantly differ between *Hif1a^ΔOB^* mice and controls (**Figure 1D**). The relative weight loss in *Vhl^ΔOB^* mice significantly differed from that of controls at early time points (**Figure 1E**), though the differences were modest. To account for potential sex-dependent differences, parallel data were obtained for male mice, and no significant differences were observed for day 14 post-infection bacterial burdens or post-infection weights (**Figure S1**). Importantly, in the data obtained in male *Vhl^ΔOB^* mice and controls, the weights did not differ from Cre-negative littermate controls at early time points.

Because the weights statistically differed at early time points in female *Vhl^ΔOB^* mice, bacterial burdens were investigated at additional time points post-infection to determine if transient differences in antibacterial responses were present. Post-infection days 3 and 5 were selected because these days represent critical times for control of bacterial burdens in prior studies in our laboratory (Putnam et al., 2019). At both days 3 and 5 post-infection, the bacterial burdens in *Vhl^ΔOB^* mice did not differ from controls (**Figure 1C**), suggesting that no substantial differences exist for bacterial burden control in these mice. Combined, the data from CFU enumeration and post-infection weights suggest that conditional knockout of *Hif1a* or *Vhl* in osteoblast-lineage cells does not substantially alter bacterial burdens in this model of *S. aureus* osteomyelitis.

Given the absence of differences in bacterial burdens when delivering doxycycline until postnatal age 4 weeks, we investigated bacterial burdens in mice with an extended washout of doxycycline. Consistent with prior publications (Wang et al., 2007; Dirckx et al., 2018), *Vhl^ΔOB^* mice with doxycycline withdrawal at postnatal age 1 week demonstrated greater baseline differences in trabecular bone compared to *Vhl^ΔOB^* mice with doxycycline withdrawal at postnatal age 4 weeks (**Figure S2**). These data support the prior studies and confirm the expected phenotype of the conditional mutants. To test the impact of bacterial burdens on mice with an extended washout of doxycycline, doxycycline was withdrawn at postnatal age 1 week, and infections took place at postnatal age 7-8 weeks. Similar to findings with withdrawal of doxycycline at postnatal age 4 weeks, no differences in bacterial burdens were observed in infected femurs of mice with withdrawal of doxycycline at postnatal age 1 week (**Figure S3A, C, E**). Similarly, no differences were observed in post-infection weights over the course of the infection (**Figure S3B, D, F**). Thus, further studies focused on doxycycline administration until postnatal age of 4 weeks to limit impacts on baseline bone volume.

### Osteoblast-lineage knockout of *Vhl* limits trabecular bone loss during *S. aureus* osteomyelitis

**Figure 2:**
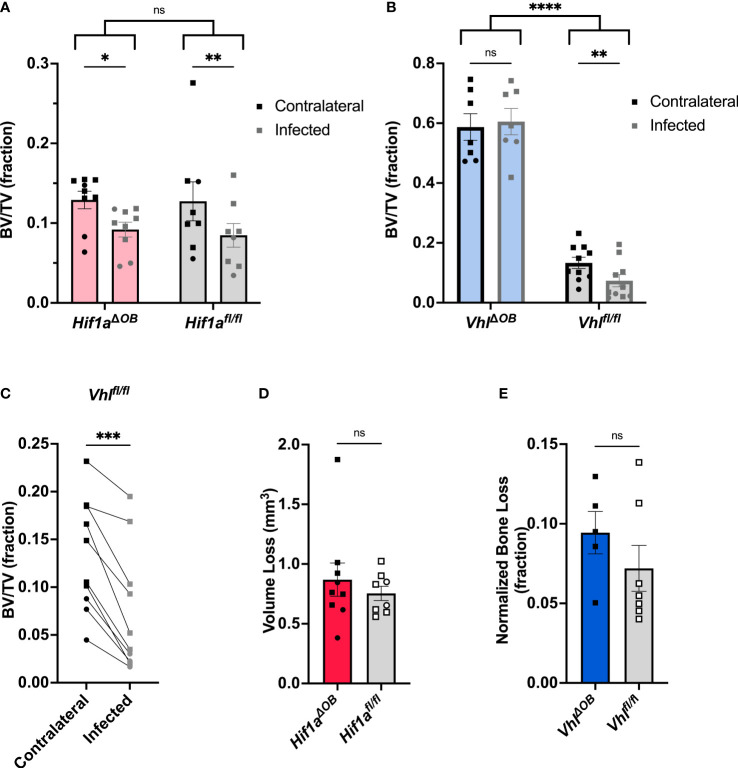
*Vhl^ΔOB^* mice do not lose trabecular bone volume during osteomyelitis. **(A)** Trabecular bone volume per total volume (BV/TV) in *Hif1a^ΔOB^* mice and *Hif1a^fl/fl^* littermate control mice (*n*=8-9) was measured in the distal metaphysis of infected and contralateral femurs using microCT. **(B)** BV/TV was measured in the infected and contralateral distal metaphyses of femurs in *Vhl^ΔOB^* mice and *Vhl^fl/fl^* littermate control mice (*n*=7-10). Squares represent males, and circles represent females; *p < 0 .05, **p < 0.01, and ns denotes not significant (*p* > 0.05) as determined by two‐way analysis of variance (ANOVA) **(A, B)**. Comparison of bracketed genotypes refers to two-way ANOVA comparison of genotypes as a whole without multiple comparisons by laterality of femur, denoted with ****p < 0.0001. **(C)** The data from *Vhl^fl/fl^* littermate control mice (*n*=10) analyzed in **(B)** are shown in an enlarged graph with lines denoting paired contralateral and infected femurs. ***p < 0.001, as determined by paired, two-tailed Student’s *t*-test. **(D)** Volume of cortical bone loss in the infected femur in *Hif1a^ΔOB^* or *Hif1a^fl/fl^* littermate control mice (*n*=8) was determined using microCT at post-infection day 14. Squares represent males, and circles represent females. **(E)** Cortical bone loss at post-infection day 14 in the infected femurs of *n*=5-7 male *Vhl^ΔOB^* or *Vhl^fl/fl^* littermate control mice was determined using microCT and is expressed as a normalized ratio relative to equivalent analysis performed on the contralateral femur. ns denotes not significant (*p* > 0.05) as determined by unpaired, two-tailed Student’s *t*-test **(D, E)**. Error bars represent mean ± SEM.

After finding that the bacterial burdens of osteoblast-lineage conditional knockout mice did not differ from those of controls, we tested the impact of osteoblast-lineage deletion of *Hif1a* on changes to bone architecture incurred during infection. To do this, we performed microCT on infected femurs and the corresponding uninfected, contralateral femurs. In previous studies, we have observed osteoclast-mediated changes in trabecular bone volume between the infected and contralateral femurs in response to *S. aureus* osteomyelitis (Putnam et al., 2019). The distal metaphyseal trabecular bone was therefore analyzed to determine the bone volume per total volume (BV/TV). In *Hif1a^ΔOB^* mice, the trabecular BV/TV did not differ from those of genotypic controls. Consistent with prior studies, the infection-induced decline in BV/TV was present in both the *Hif1a^ΔOB^* mice as well as the Cre-negative littermate controls (**Figure 2A**). While BV/TV was not different between *Hif1a^ΔOB^* mice and Cre-negative littermate controls, trabecular BV/TV was markedly increased in *Vhl^ΔOB^* mice compared to Cre-negative littermate controls (**Figure 2B**). The dramatic increase in trabecular BV/TV has been reported previously and supports the appropriate activity of the Cre-recombinase (Wang et al., 2007; Dirckx et al., 2018). Furthermore, when measuring changes in BV/TV in response to infection, no difference in trabecular bone loss was observed relative to the contralateral limb in *Vhl^ΔOB^* mice (**Figure 2B**). The Cre-negative littermate control group demonstrated significant loss of BV/TV during infection between the infected and contralateral femurs (**Figure 2C**). Representative cross-sections through the distal metaphysis are shown in **Figure S4A** and include both the cortical shell and the internal trabecular bone that is analyzed for trabecular BV/TV. These data suggest that loss of osteoblast-lineage *Hif1a* does not impact *S. aureus*-mediated changes in trabecular BV/TV during infection, but loss of osteoblast-lineage *Vhl* limits infection-mediated decreases in trabecular BV/TV during *S. aureus* osteomyelitis.

In addition to analyzing changes in trabecular bone, cortical bone loss was also measured by microCT. As shown in **Figure S4A**, cortical bone thinning was present in both *Vhl^ΔOB^* mice and *Vhl^fl/fl^* mice. However, measurement of cortical bone loss in *Vhl^ΔOB^* mice was more difficult compared to analyses of control mice. Prior studies have identified that cortical bone is trabecularized (with gaps in the cortical shell) along the diaphysis in *Vhl^ΔOB^* mice, most prominently near the metaphyses (Dirckx et al., 2018). Therefore, methods for cortical bone analysis were altered for this genotype to limit the potential falsely elevated measurement of cortical bone lysis caused by trabecularized cortical bone. Namely, the area of analysis for cortical bone lysis was confined to a central region of the diaphysis that excluded the metaphyseal area where trabecularized cortical bone is most prevalent. The cortical bone analysis was performed on the equivalent region of the contralateral femur. The value of the measured bone trabecularization on the contralateral limb was then used to normalize the measured lysis in the infected limb. This modification was used for both the *Vhl^ΔOB^* mice and the *Vhl^fl/fl^* littermate control mice. MicroCT reconstructions of representative femurs from *Vhl^ΔOB^* mice and controls are shown (**Figure S4B**). For the standard analysis in the *Hif1a^ΔOB^* mice and for modified analysis in the *Vhl^ΔOB^* mice, cortical lysis was not found to significantly differ between the conditional knockout mice and their Cre-negative littermate controls, respectively (**Figures 2D, E**).

Recognizing that injury itself may contribute to trabecular bone loss, we investigated changes in trabecular BV/TV following sterile injury (mock infection). Animals were subjected to the same procedure used to induce osteomyelitis except that sterile PBS was injected into the medullary space instead of *S. aureus*. Following mock infection, no changes were observed in the trabecular BV/TV between the injured and contralateral femurs of *Vhl^ΔOB^* mice (**Figure S5**). Similarly, no changes were observed between the injured and contralateral femurs of Cre-negative littermate control mice (**Figure S5**). These data suggest that trabecular bone loss occurs to a greater extent in infected femurs compared to mock-infected femurs.

### Conditional knockout of *Vhl* in osteoblast-lineage cells limits infection-induced increases in osteoclasts *in vivo* and alters RANKL-OPG signaling *in vitro*

**Figure 3:**
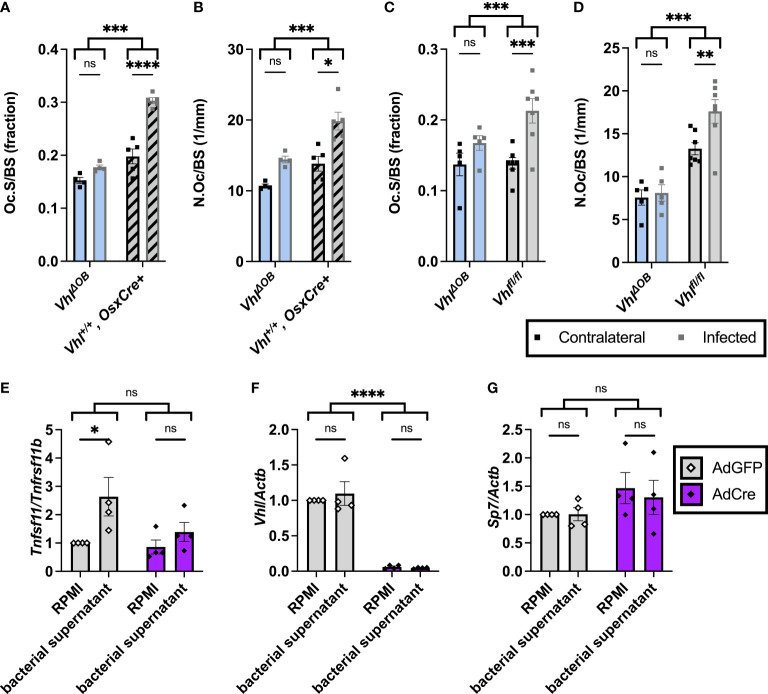
Fewer osteoclasts are present in trabecular bone of *Vhl^ΔOB^* mice during osteomyelitis. **(A)** Bone histomorphometry was performed on trabecular bone in the distal metaphysis of infected and contralateral femurs for male *Vhl^ΔOB^* mice (*n*=4) and *Vhl^+/+^, OsxCre+* mice (age-matched, *n*=4) in order to quantify osteoclast surface per bone surface (Oc.S/BS), expressed as a fraction. ***p < 0.001, ****p < 0.0001, and ns denotes not significant (*p* > 0.05) as determined by two‐way analysis of variance (ANOVA) with correction for multiple comparisons. **(B)** Equivalent analysis of sections analyzed in **(A)** were performed to measure the number of osteoclasts per bone surface (N.Oc/BS). **(C, D)** In an independent experiment evaluating male *Vhl^ΔOB^* mice (*n*=5), littermate *Vhl^fl/fl^* (Cre-negative) control mice (*n*=7) were used instead of the age-matched control mice to evaluate Oc.S/BS **(C)** and N.Oc/BS **(D)**. *p < 0.05, **p < 0.01, ***p < 0.001, ****p < 0.0001, and ns denotes not significant (*p* > 0.05) as determined by two‐way ANOVA with correction for multiple comparisons **(A-D)**. Straight lines refer to comparison between contralateral and infected femurs in each genotype; comparison between bracketed groups refers to two-way ANOVA comparison of genotypes as a whole without multiple comparisons by laterality of femur. **(E-G)** *Vhl^fl/fl^* primary osteoblasts were treated with AdCre or AdGFP (vehicle control) to induce knockout of *Vhl*. Following manipulation with AdCre or AdGFP, cells were treated with toxin-deficient (Δ*psm*/Δ*spa* USA300 *S. aureus*) bacterial supernatant or RPMI (vehicle control). **(E)** Fold change in *Tnfsf11* (RANKL) is shown relative to *Tnfrsf11* (OPG) using the 2^-ΔΔ^ *^C^* ^T^ method and normalized to the RPMI-AdGFP condition. Fold changes in transcription of *Vhl* **(F)** and *Sp7* (Osx) **(G)** relative to the control gene *Actb* are shown as determined by the 2^-ΔΔ^ *^C^* ^T^ method and normalized to the RPMI-AdGFP condition. *p < 0.05, ****p <0 .0001, and ns denotes not significant (*p* >0.05) as determined by two‐way ANOVA of the log-transformed fold changes in gene expression **(E-G)**. Straight lines refer to comparison between RPMI and bacterial supernatant in each adenovirus-treated group; bracketed groups refer to two-way ANOVA comparison between AdGFP- and AdCre-treated cells as a whole without multiple comparisons by specific bacterial treatment groups. *n*=4 independent replicates, each with 3 technical replicates of cells. AdGFP, adenovirus containing GFP; and AdCre, adenovirus containing Cre recombinase and GFP. Error bars represent mean ± SEM.

Previous studies revealed that trabecular bone loss during *S. aureus* osteomyelitis is associated with an increase in osteoclasts in trabecular bone (Putnam et al., 2019). To better understand why trabecular BV/TV loss is decreased in infected *Vhl^ΔOB^* mice, we performed histomorphometry on bone sections from infected mice to measure the abundance of osteoclasts. *Vhl^ΔOB^* mice demonstrated a decreased number of osteoclasts and osteoclast surface per bone surface compared to controls (age-matched *OsxCre*+ mice lacking the *Vhl* floxed alleles and *Vhl^fl/fl^* littermate control mice) (**Figures 3A–D**). Representative images of TRAP-stained trabecular bone in infected and contralateral femurs of *Vhl^ΔOB^* mice and *Vhl^fl/fl^* littermate control mice are shown in **Figure S6**. Loss of *Vhl* in the osteoblast lineage therefore limits osteoclast abundance in trabecular bone during *S. aureus* osteomyelitis *in vivo*.

Conditional deletion of negative regulators of HIF signaling upstream of VHL has previously been shown to alter the receptor activator of NF-κB ligand (RANKL)-osteoprotegerin (OPG) signaling axis (Wu et al., 2015). One of the primary ways that osteoblast-lineage cells impact osteoclast abundance is through this RANKL-OPG signaling axis (Teitelbaum, 2000). During *S. aureus* osteomyelitis, osteoblast RANKL is increased, which facilitates bone loss (Mbalaviele et al., 2017). We previously found that *S. aureus* supernatants are potently cytotoxic to osteoblasts, and this cytotoxicity is dependent on α-type phenol-soluble modulins (PSMα) (Cassat et al., 2013). To understand if the RANKL-OPG signaling axis impacts differences in observed osteoclast numbers during *S. aureus* osteomyelitis, *in vitro* studies were performed in which osteoblasts were treated with toxin-deficient *S. aureus* supernatants that lack the α-type PSMs. To investigate how loss of *Vhl* in osteoblast-lineage cells modifies RANKL activity relative to that of OPG, we used an adenovirus vector to deliver Cre recombinase to *Vhl^fl/fl^* osteoblasts. Adenoviruses containing green fluorescent protein (AdGFP, vehicle control) or Cre recombinase and GFP (AdCre) were used to transfect *Vhl^fl/fl^* osteoblasts *in vitro* (Dirckx et al., 2018). Transfection with AdCre causes deletion of the floxed *Vhl* exon resulting in knockout. Following transfection with AdGFP or AdCre, osteoblasts were stimulated with toxin-deficient bacterial supernatants. Reverse transcription-polymerase chain reaction (RT-PCR) was used to assess changes in *Tnfsf11* (RANKL) transcription relative to *Tnfrsf11b* (OPG) transcription. When analyzed by multiple comparisons with correction, toxin-deficient supernatants from *S. aureus* significantly increased the transcription of the gene for RANKL relative to that of OPG in the AdGFP group but not in the AdCre group in which *Vhl* is knocked out (**Figure 3E**). By two-way ANOVA, the comparison between AdGFP and AdCre groups was analyzed without discrimination between the bacterial treatment subsets and had a *p*-value of 0.0549 (**Figure 3E**). In these experiments, transcription of *Vhl* was also analyzed to confirm that Cre-mediated deletion significantly reduced *Vhl* transcription (**Figure 3F**). Moreover, *Sp7* (Osx) transcription was transcribed in all conditions and did not differ between groups (**Figure 3G**). Taken together, these data suggest that *Vhl* deletion in osteoblasts diminishes *S. aureus*-mediated increases in the transcription of *Tnfsf11* (RANKL) relative to *Tnfsf11b* (OPG).

### Conditional knockout of *Vhl* or *Hif1a* in myeloid cells does not alter bacterial burdens during osteomyelitis

To better understand the impact of HIF signaling in response to *S. aureus* osteomyelitis, we next focused on the myeloid lineage, which gives rise to bone-resorbing osteoclasts and innate immune cells such as neutrophils and macrophages. To test the impact of myeloid cell HIF signaling on antibacterial responses and maintenance of bone architecture during *S. aureus* osteomyelitis, a second set of conditional knockout mice was generated. Using *LysMCre* and the corresponding floxed *Hif1a^fl/fl^* and *Vhl^fl/fl^* alleles, myeloid-lineage conditional knockouts of *Hif1a* (*Hif1a^fl/fl^*, *LysMCre^+/-^*; *Hif1a^ΔMyeloid^*) and *Vhl* (*Vhl^fl/fl^*, *LysMCre^+/-^*; *Vhl^ΔMyeloid^*) were generated and subjected to *S. aureus* osteomyelitis. Unlike the *OsxCre* mouse, this *LysMCre* model does not incorporate a repressor or inducer. Therefore, the Cre recombinase is active throughout *in utero* and postnatal development. Because the *LysMCre*-mediated conditional knockout mice do not exhibit a substantial bone phenotype that confirms Cre activity (data not shown), bone marrow-derived macrophages were isolated from *Vhl^ΔMyeloid^* mice and control mice. *Vhl* transcript levels were measured to compare the efficiency of the Cre-mediated knockout. *Vhl^ΔMyeloid^* mice exhibited significantly decreased (77.4% mean reduction, 95% confidence interval of 61.0-87.9%) *Vhl* mRNA transcript levels compared to those from cells of control mice, confirming the efficient activity of the Cre recombinase with the *LysMCre* model (**Figure S7**).

**Figure 4:**
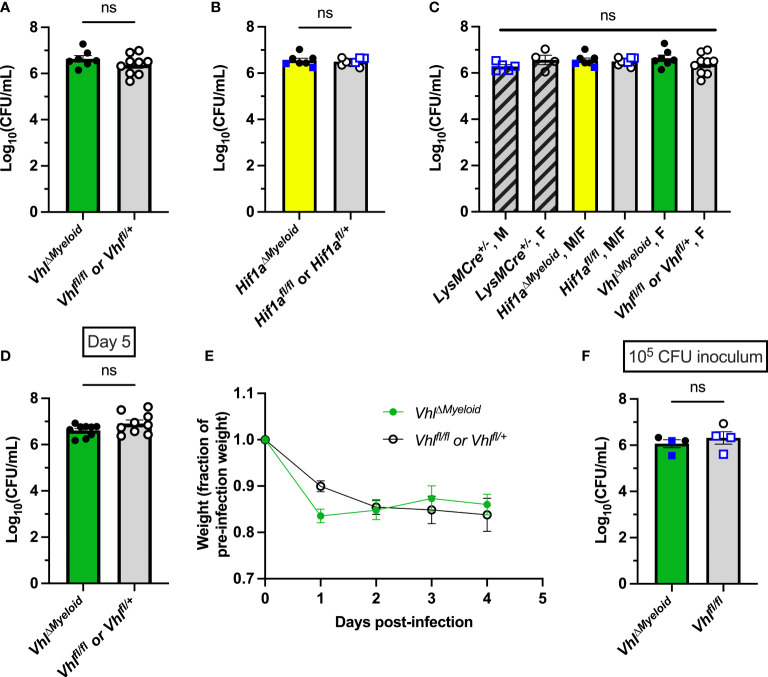
Myeloid-lineage conditional knockout of *Hif1a* or *Vhl* does not alter bacterial burdens during *S. aureus* osteomyelitis. **(A)** Day 14 post-infection bacterial burdens from infected femurs of female *Vhl^ΔMyeloid^* mice or Cre-negative controls composed of both *Vhl^fl/fl^* and *Vhl^fl/+^* littermates (*n*=7-9). **(B)** Day 14 post-infection bacterial burdens from infected femurs of *Hif1a^ΔMyeloid^* mice and Cre-negative controls, which are composed of both *Hif1a^fl/fl^* and *Hif1a^fl/+^* littermates. Mice (*n*=7-8) are female except for those marked with blue squares, which represent data from male mice. **(C)** Day 14 post-infection bacterial burdens from infected femurs of male and female (as labeled) age-matched *LysMCre^+/-^* mice (*n*=4) that do not contain floxed alleles are shown along with the data displayed in **(A, B)** for comparison between all groups. **(D)** Bacterial burdens were determined in the infected femurs of *Vhl^ΔMyeloid^* mice and Cre-negative littermate controls (*Vhl^fl/fl^* or *Vhl^fl/+^*) at day 5 post-infection (*n*=9). **(E)** The relative post-infection weights corresponding to the experiment shown in **(D)** are shown relative to the pre-infection starting weight; (*n*=4-5 from first half of mice in the experiment—when weights were measured daily). Relative post-infection weights do not differ between genotypes as compared by two-way analysis of variance (ANOVA). **(F)** Bacterial burdens were determined in the infected femurs of *Vhl^ΔMyeloid^* and Cre-negative littermate controls (*Vhl^fl/fl^*) at day 14 post-infection following infection with a lower inoculum of 10^5^ CFU. Error bars represent mean ± SEM, and ns denotes difference did meet the *p* < 0.05 standard for significance as determined by unpaired, two-tailed Student’s *t*-tests **(A, B, D, F)** or one-way ANOVA **(C)**. CFU=colony forming units.

Bacterial burdens in *Hif1a^ΔMyeloid^* and *Vhl^ΔMyeloid^* mice and their respective controls were assessed to understand the impact of myeloid HIF-1α and VHL on antibacterial responses. As observed with conditional knockout in the osteoblast lineage, bacterial burdens at post-infection day 14 did not differ between *Hif1a^ΔMyeloid^* and *Vhl^ΔMyeloid^* mice and their respective control mice (**Figures 4A–C**). These data further support that myeloid-lineage conditional knockout of *Hif1a* or *Vhl* does not substantially alter bacterial clearance at day 14 post-infection.

Prior studies have shown that *Vhl* inactivation improves myeloid cell longevity and bacterial phagocytosis (Walmsley et al., 2006). Therefore, we further explored bacterial burdens at earlier time points when phagocytes are interacting with *S. aureus* at the time of abscess formation. In prior studies, we have shown that post-infection day 5 coincides with bacterial control in our model (Putnam et al., 2019). Therefore, bacterial burdens at day 5 post-infection were measured to determine if transient changes occurred early during infection. Bacterial burdens were not found to differ between *Vhl^ΔMyeloid^* mice and Cre-negative littermate controls at day 5 post-infection (**Figure 4D**). Moreover, the weights at the early timepoints during this study did not significantly differ, further supporting the notion that no meaningful differences in systemic infection responses occurred (**Figure 4E**). In a subsequent experiment, a reduced bacterial inoculum (10^5^ CFU) was delivered with the hypothesis that an inoculum of 10^6^ CFU may overwhelm antibacterial responses. Bacterial burdens between *Vhl^ΔMyeloid^* mice and Cre-negative littermate control mice did not differ with the lower inoculum, indicating that *Vhl* conditional knockout in the myeloid lineage does not significantly alter bacterial clearance during *S. aureus* osteomyelitis (**Figure 4F**). Overall, these data suggest that conditional deletion of *Hif1a* or *Vhl* in myeloid cells does not affect bacterial burdens during osteomyelitis.

### Conditional knockout of *Vhl* in myeloid cells enhances bone loss during *S. aureus* osteomyelitis

**Figure 5:**
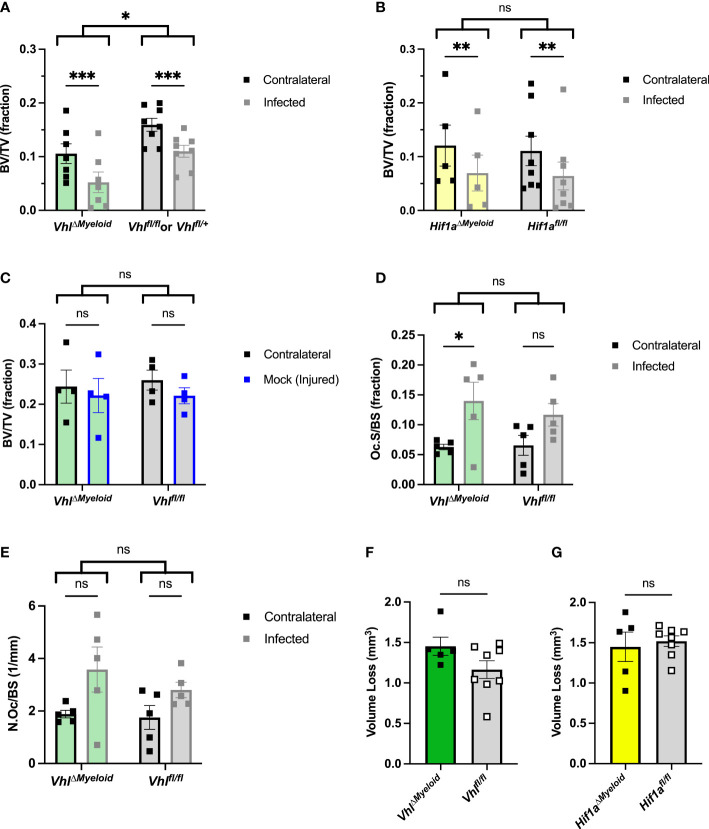
Conditional knockout of *Vhl* in myeloid cells slightly decreases trabecular bone volume during *S. aureus* osteomyeltis. **(A)** Trabecular bone volume per total volume (BV/TV) in *Vhl^ΔMyeloid^* mice and Cre-negative littermate *Vhl^fl/fl^* or *Vhl^fl/+^* control mice (*n*=7-8, male) at post-infection day 14 was measured in the distal metaphysis of infected and contralateral femurs. The differences between the groups were compared by two-way analysis of variance (ANOVA), which revealed that the genotypes (two bracketed groups) significantly differ. **(B)** Trabecular BV/TV in *Hif1a^ΔMyeloid^* mice and littermate *Hif1a^fl/fl^* control mice (*n*=5-8, male) at post-infection day 14 was measured in the distal metaphysis of infected and contralateral femurs. The genotypes (two bracketed groups) did not significantly differ (ns) as compared by two-way ANOVA. **(C)** The BV/TV in *Vhl^ΔMyeloid^* mice and littermate *Vhl^fl/fl^* control mice (*n*=4, male) at post-injury day 14 was measured in the trabecular bone of the distal metaphysis of mock-infected and contralateral femurs. Injury significantly reduced BV/TV compared to contralateral femurs (*p* < 0.05, not depicted; ns by multiple comparisons within genotypes by two-way ANOVA with correction for multiple comparisons). The genotypes (bracketed groups) did not significantly differ as compared by two-way ANOVA. **(D)** Osteoclast surface per bone surface (Oc.S/BS) is expressed as a fraction from bone histomorphometry performed on trabecular bone in the distal metaphysis of infected and contralateral femurs for male *Vhl^ΔMyeloid^* mice (*n*=5) and Cre-negative littermate *Vhl^fl/fl^* mice (*n*=5). The measurements for the infected and contralateral femurs are statistically different (*p* < 0.01), and the genotypes do not significantly differ by two‐way ANOVA. With correction for multiple comparisons, the measurements of the infected and contralateral limbs do not reach significance for the Cre-negative littermate but do for the *Vhl^ΔMyeloid^* mice. **(E)** Parallel analysis of sections analyzed in **(D)** were performed to measure the number of osteoclasts per bone surface (N.Oc/BS). The measurements for the infected and contralateral limbs are statistically different (*p* < 0.05), and the genotypes do not significantly differ by two‐way ANOVA. With correction for multiple comparisons, the measurements of the infected and contralateral limbs do not reach significance. **(F, G)** The volume of cortical bone loss in male *Vhl^ΔMyeloid^* **(F)** or *Hif1a^ΔMyeloid^* **(G)** mice and respective littermate controls at post-infection day 14 was assessed by microcomputed tomography (*n*=5-8). *p < 0.05, **p < 0.01, ***p < 0.001, and ns denotes difference did not meet the *p* < 0.05 standard for significance as determined by two‐way ANOVA with correction for multiple comparisons of the infected (or mock infected) and contralateral limbs for each genotype individually **(A-D, G)**, or as determined by unpaired, two-tailed Student’s *t*-test **(E, F)**. *P*-values between bracketed groups of data refer to two-way ANOVA assessment of genotype as a variable without multiple comparisons by laterality (infected vs. contralateral) of femur **(A-D, G)**. Error bars represent mean ± SEM.

After confirming that bacterial burdens do not significantly differ during *S. aureus* osteomyelitis following genetic ablation of *Hif1a* or *Vhl* in the myeloid lineage, we next tested the impact of these conditional knockouts on pathologic changes to bone architecture. Active HIF signaling in myeloid-lineage cells has been shown to increase proinflammatory (and pro-osteoclastogenic) cytokines in other models of inflammation (Nizet and Johnson, 2009; Mbalaviele et al., 2017). Thus, we investigated the impact of myeloid-lineage conditional knockouts of *Hif1a* or *Vhl* on pathologic changes to bone architecture using microCT at day 14 post-infection. In *Vhl^ΔMyeloid^* mice, average BV/TV in the trabecular bone of both femurs was decreased compared to trabecular BV/TV in both femurs of Cre-negative littermate control mice (**Figure 5A**). In addition to assessing genotype as a variable by two-way ANOVA, BV/TV of the infected limbs was compared to that of the contralateral limbs as the second variable of the ANOVA. We observed a significant decrease in BV/TV in infected femurs relative to that of contralateral femurs in both *Vhl^ΔMyeloid^* and Cre-negative littermate control mice (**Figure 5A**). In *Hif1a^ΔMyeloid^* mice, BV/TV in trabecular bone did not differ between the *Hif1a^ΔMyeloid^* mice and the Cre-negative littermate controls by genotype, and again, the data showed a significant decrease in BV/TV in the infected femurs of both genotypes relative to the contralateral femur (**Figure 5B**). For *Hif1a^ΔMyeloid^* mice, female mice were also assessed and showed similar results to those of the males (**Figure S8**). Therefore, conditional knockout of *Hif1a* does not impact trabecular bone changes. On the contrary, *Vhl^ΔMyeloid^* mice have a modest, yet significant, reduction in trabecular BV/TV compared to that of Cre-negative littermate controls following *S. aureus* osteomyelitis.

To better understand why differences in trabecular BV/TV exist between *Vhl^ΔMyeloid^* mice and Cre-negative littermate control mice following infection, animals were mock infected and analyzed for changes in BV/TV after 14 days. In the mock-infected mice, the differences in BV/TV between genotypes were not present, unlike observations in the setting of infection (**Figure 5C**). The injury itself in mock-infected animals caused a significant difference in trabecular BV/TV compared to the contralateral limbs by two-way ANOVA; although, this difference was not present within genotypes by multiple comparisons (**Figure 5C**). Another possible reason for decreases in trabecular BV/TV in infected *Vhl^ΔMyeloid^* mice relative to BV/TV measurements in control mice could be increased inflammation at the site of infection. To test this hypothesis, cytokine abundance was determined in infected and contralateral femurs of *Vhl^ΔMyeloid^* mice and control animals at post-infection day 5. Day 5 was chosen because it coincides with achievement of bacterial burden control in wild-type mice as published previously, and these time points correspond with high cytokine levels (Putnam et al., 2019). No differences in major cytokines were detected (**Figure S9**). In particular, levels of TNF-α and IL-1β, two cytokines known to enhance osteoclast formation, were not significantly different from those of control animals. Despite no differences between the genotypes, the cytokines generally demonstrated significant increases in the infected femurs compared to the contralateral femurs (**Figure S9**). Thus, the differences in trabecular BV/TV between *Vhl^ΔMyeloid^* mice and Cre-negative littermate control mice do not correspond to alterations in cytokines abundances at post-infection day 5. Finally, no genotype-specific differences were observed when histomorphometry was performed to analyze osteoclast surface per bone surface (**Figure 5D**) or the number of osteoclasts per bone surface (**Figure 5E**).

In addition to analyzing infection-mediated bone loss in trabecular bone, cortical bone loss was also analyzed in *Hif1a^ΔMyeloid^* and *Vhl^ΔMyeloid^* mice. The volume of cortical bone loss in *Vhl^ΔMyeloid^* mice and Cre-negative littermate controls was not significantly different (**Figure 5F**). Conditional knockout of *Hif1a* in the myeloid lineage also does not alter cortical bone loss during *S. aureus* osteomyelitis (**Figure 5G**). Thus, *Hif1a* conditional knockout in the myeloid lineage does not impact bacterial burdens or pathologic changes in bone architecture; however, trabecular BV/TV in *Vhl^ΔMyeloid^* mice is significantly lower at post-infection day 14 compared to the trabecular bone of Cre-negative littermate control mice.

## Discussion

Osteomyelitis is a complex disease that involves many different cell types and signaling pathways that regulate bone architecture and immune responses. HIF signaling is critical during bone development, and augmentation of HIF signaling is known to improve bone regeneration following injury (Wang et al., 2007; Wan et al., 2008). Furthermore, HIF signaling is intricately linked to innate immune responses and has been proposed as a therapeutic target during infection (Nizet and Johnson, 2009). While studied in the context of other infection models (Wickersham et al., 2017), the role of HIF signaling during osteomyelitis is unknown despite interest in the modulation of HIF signaling for both bone repair and infection treatment, independently (Wan et al., 2008; Bhandari and Nizet, 2014). In this study, two different Cre recombinase mouse strains (*OsxCre* and *LysMCre*) were used to target osteoblast- and myeloid-lineage cells, respectively, to determine the cell-specific contributions of HIF signaling during osteomyelitis (Clausen et al., 1999; Rodda and McMahon, 2006; Chen et al., 2014). *Hif1a^fl/fl^* and *Vhl^fl/fl^* mice were crossed with these Cre strains to generate mice with conditional knockout of *Hif1a* or *Vhl* in either the osteoblast or myeloid lineages. These two Cre models target the lineages that give rise to the canonical cell types present in bone: osteoblasts/osteocytes (osteoblast lineage) and osteoclasts (myeloid lineage). In addition to targeting osteoclasts, *LysMCre* targets innate immune cells such as granulocytes and macrophages, cells in which HIF signaling has been shown to be essential for inflammatory responses (Cramer et al., 2003).

Conditional knockout of *Hif1a* in both the osteoblast and myeloid lineages does not impact bacterial burdens or infection-associated changes in bone architecture, suggesting that *Hif1a* does not contribute to pathogenesis during *S. aureus* post-traumatic osteomyelitis. It is possible that HIF-1α plays an important role in bacterial clearance in other models of osteomyelitis or that differences may exist at time points not investigated in this study. However, it has also been suggested that, despite distinct gene targets between HIF-1α and HIF-2α, the overlap of large sets of target genes may allow HIF-2α to compensate for many, though not all, HIF-1α activities (Wang et al., 2007; Kaelin and Ratcliffe, 2008). To explore this hypothesis, it will be important to test a double knockout of *Hif1a* and *Epas1* (the gene encoding HIF-2α) or a knockout of the gene for the joint heterodimer *Arnt* to better understand the impact of loss of global HIF signaling during osteomyelitis and not just HIF-1α. Direct measurement of HIF-2α by immunohistochemistry in mice lacking *Hif1a* may also provide insight into such compensation.

MicroCT analysis of trabecular and cortical bone revealed no differences in bone changes during *S. aureus* infection following conditional knockout of *Hif1a* in both cell lineages tested. Prior bone phenotypes following osteoblast-lineage knockout of *Hif1a* have revealed relatively subtle phenotypes, which may not have been readily detected in these studies given the potentially overwhelming influence of infection in our model. The role of HIF-target VEGF is dependent upon the skeletal model, and impacts on VEGF signaling downstream of *Hif1a* deletion may have been masked by use of only one animal model (Buettmann et al., 2019). However, given the data regarding bacterial burdens and microCT findings from these studies, osteoblast- and myeloid-lineage HIF-1α are not essential for control of *S. aureus* osteomyelitis.

Conditional knockout of *Vhl* in osteoblast versus myeloid lineages causes different phenotypes in the trabecular bone following infection. Conditional knockout of *Vhl* in the osteoblast lineage causes a profound increase in trabecular BV/TV, as observed previously (Wang et al., 2007; Dirckx et al., 2018). Unlike Cre-negative control mice, *Vhl^ΔOB^* mice do not demonstrate a decline in BV/TV in infected bone, suggesting that deletion of *Vhl* in osteoblast-lineage cells alters bone homeostasis and limits pathologic bone loss during infection. Histomorphometry showed that, unlike in trabecular bone of control mice, osteoclast abundance measurements do not increase in the infected femur of *Vhl^ΔOB^* mice following infection. *In vitro*, deletion of *Vhl* in osteoblasts decreases transcription of the gene for RANKL relative to that for OPG, indicating that *Vhl* deletion in osteoblasts may regulate osteoclast formation through impacts on the RANKL/OPG ratio. Functional *in vitro* studies that test osteoclast formation in co-culture with osteoblasts lacking *Vhl* will help to establish if *Vhl* deletion in osteoblasts impacts osteoclastogenesis during infection. While alternative mechanisms may also impact osteoclast formation *in vivo*, these data suggest *Vhl* deletion in osteoblasts limits infection-induced RANKL production relative to OPG and limits loss of trabecular bone. It will be important to directly test this hypothesis in future studies. The mechanism by which *Vhl* deletion alters the RANKL/OPG ratio is unknown. Prior studies investigating HIF-1α have not directly linked HIF signaling to RANKL production (Wu et al., 2015). Investigation of HIF-2α in osteoblast-lineage cells has suggested that HIF-2α activation increases RANKL production and osteoclast formation (Lee et al., 2019). However, this phenotype was not replicated in another study, which instead found that HIF-2α increases OPG production and limits osteoclast formation (Wu et al., 2015). The findings of our study are consistent with a prior investigation that found *Vhl* knockout in osteoblasts inhibited osteoclast formation in co-culture and decreased RANKL gene expression (Wang et al., 2012). Conditional deletion of *Vhl* in osteoblasts has also been shown to inhibit osteoclasts through increased OPG and through a microRNA-IL-33-Notch1 signaling axis, which may represent an alternative mechanism (Kang et al., 2017). Independent of HIF signaling, VHL interacts with several other proteins that may impact the responses of osteoblasts to *S. aureus* stimulation (Li and Kim, 2011). To better understand if HIF signaling mediates the phenotypic changes in trabecular bone in *Vhl^ΔOB^* mice, double knockout mice lacking *Vhl* along with *Hif1a*, *Epas1*, or *Arnt* could be tested. Notably, prior studies have investigated the direct activities of *Hif1a* and *Vhl* deletion in osteoblast-lineage cells on osteogenesis itself (Wang et al., 2007; Regan et al., 2014; Amir et al., 2022). During osteomyelitis and inflammatory osteolysis, osteoclast-mediated resorption is proposed to be the primary mechanism of bone loss (Mbalaviele et al., 2017), which was the rationale for the primary outcome explored in these studies.

While *Vhl^ΔOB^* mice do not display infection-mediated reduction in trabecular BV/TV, *Vhl^ΔMyeloid^* mice exhibit BV/TV values slightly lower than those of Cre-negative control mice, indicative of increased bone loss. The contralateral femurs show a similar decline in trabecular BV/TV relative to that of littermate control mice, indicating that trabecular bone homeostasis might be disturbed systemically during infection. By direct measurement of osteoclast surface and number per bone surface, no significant differences between *Vhl^ΔMyeloid^* mice and controls were observed. However, resorptive capacity may still differ, or transient differences may have been missed. Furthermore, these data do not directly support the hypothesis that *Vhl^ΔMyeloid^* mice exhibit increased osteoclastogenesis in association with increased trabecular bone loss.

There are multiple limitations to the studies performed. While most experiments were performed in male and female mice, not all studies were powered to draw sex-specific conclusions, though generally the data do not support a dramatic difference between the sexes. These studies explored a single model of osteomyelitis, and alternative models may reveal different findings. Additionally, only discrete time points were selected up to 14 days and transient phenotypes may have been missed as well as phenotypes dependent upon chronic infection exceeding the 14-day experiment. The mechanisms by which *Vhl* deletion in osteoblast and myeloid cells alter trabecular bone loss have not been fully established, especially in the context of infection, and future studies should explore the role of HIF signaling on osteoblast anabolic activities during infection. Moreover, VHL-dependent, HIF-independent mechanisms remain possible and have not been explored.

While these data lay the framework for understanding the impact of HIF signaling on antibacterial responses and bone remodeling during *S. aureus* osteomyelitis, multiple questions should be investigated through future studies. While not the intended experimental comparison for which the experiments were designed to investigate, average trabecular BV/TV in the contralateral femurs of mock-infected animals was higher than BV/TV in contralateral femurs of *S. aureus*-infected femurs. The consistency of these data suggests that a systemic response to infection alters bone homeostasis through a circulating mediator. Supporting the hypothesis that systemic inflammatory responses during *S. aureus* osteomyelitis cause trabecular bone loss, multiple diseases that trigger systemic inflammation have been associated with bone loss (Shim et al., 2018; Adami et al., 2019; Ke et al., 2019). Future studies should directly test if trabecular bone loss occurs in non-infected bones of mice infected with *S. aureus* (or other pathogens) in the bone or non-osseous sites.

Genetic studies were conducted with an interest in understanding the basic biology and potential for therapeutic intervention. Thus, future studies should investigate pharmacologic targeting of the HIF signaling pathway. Pharmacologic studies (or an inducible *Ubiquitin-Cre* model) would allow for investigation of cell agnostic manipulation of HIF signaling to understand overall cell signaling influences. Furthermore, baseline differences in *Vhl^ΔOB^* mice may substantially impact trabecular bone biology outside of cell signaling itself. Pharmacologic intervention would permit investigation of augmented HIF signaling without extreme differences in bone architecture prior to infection. The potential to target VHL (Frost et al., 2016) or HIF PHDs (Haase, 2017) will also improve understanding of HIF-dependent versus HIF-independent functions of VHL. Pharmacologic targeting of HIF during osteomyelitis could also offer therapeutic solutions such as dual-purpose copper (II) treatment that inhibits HIF PHDs and is inherently antibacterial (Wu et al., 2013; Yan et al., 2021).

Overall, these studies demonstrate that bone cell *Hif1a* does not contribute to control of bacterial burdens and pathologic changes in bone architecture during osteomyelitis. Furthermore, *Vhl* was found to impact trabecular BV/TV in response to infection in a cell-type specific manner. These findings support further investigation of the impact of HIF signaling on bone architecture during states of pathologic bone loss such as osteomyelitis.

## Data availability statement

The raw data supporting the conclusions of this article will be made available by the authors, without undue reservation

## Ethics statement

The animal study was reviewed and approved by Vanderbilt University Medical Center Institutional Animal Care and Use Committee (IACUC).

## Author contributions

CF and JC conceptualized the paper. CF and JC wrote the paper. CF, IH, and JC analyzed the data. CF, IH, JC, CP, LF, JC, TS, VV, JJ, and SP conducted experiments. All authors contributed to the article and approved the submitted version.

## Funding

JC is currently supported by the National Institute of Allergy and Infectious Diseases grants R01AI145992, R01AI161022, and R01AI132560, and was formerly supported by K08AI113107. JC is also supported by a Career Award for Medical Scientists from the Burroughs Wellcome Fund, and by the G. Harold and Leila Y. Mathers Charitable Foundation. CF was supported by the National Institute of General Medical Sciences Medical Scientist Training Program grant T32GM007347, and the National Institute of Allergy and Infectious Diseases grant F30AI138424. CP was also supported by NIH grant T32M007347 and is supported by the National Institute of Diabetes and Digestive and Kidney Diseases grant F30DK120114. TS was supported by National Institute of Arthritis and Musculoskeletal and Skin Diseases grant R01AR064772. SP and JJ were supported by funding from the Department of Medicine at Vanderbilt University Medical Center. Research support for the μCT50 and computer cluster is provided by National Institutes of Health grant S10RR027631.

## Acknowledgments

The authors would like to acknowledge the guidance and assistance of Jarrod True, Sasidhar Uppuganti, Margaret Allaman, Carlos H. Serezani, Rachelle Johnson, and members of the Cassat laboratory (past and present) not on the author list who contributed to discussions during laboratory meetings. A portion of the data and text of this manuscript first appeared in the following dissertation: Ford, C.A. (2021) The role of HIF signaling during *Staphylococcus aureus* osteomyelitis and biomaterial-based treatment strategies. [dissertation]. [Nashville (TN)]: Vanderbilt University. Available at: http://hdl.handle.net/1803/16660.

## Conflict of interest

The authors declare that the research was conducted in the absence of any commercial or financial relationships that could be construed as a potential conflict of interest.

## Publisher’s note

All claims expressed in this article are solely those of the authors and do not necessarily represent those of their affiliated organizations, or those of the publisher, the editors and the reviewers. Any product that may be evaluated in this article, or claim that may be made by its manufacturer, is not guaranteed or endorsed by the publisher.
