# Supplementary material for: Loss of Vhl alters trabecular bone loss during S. aureus osteomyelitis in a cell-specific manner
Source: Front Cell Infect Microbiol. 2022 Sep 20;12:985467. doi: 10.3389/fcimb.2022.985467 (PMC9530664; doi:10.3389/fcimb.2022.985467)
Supplement: Supplementary file 1 [file DataSheet_1.pdf]

Supplemental figures

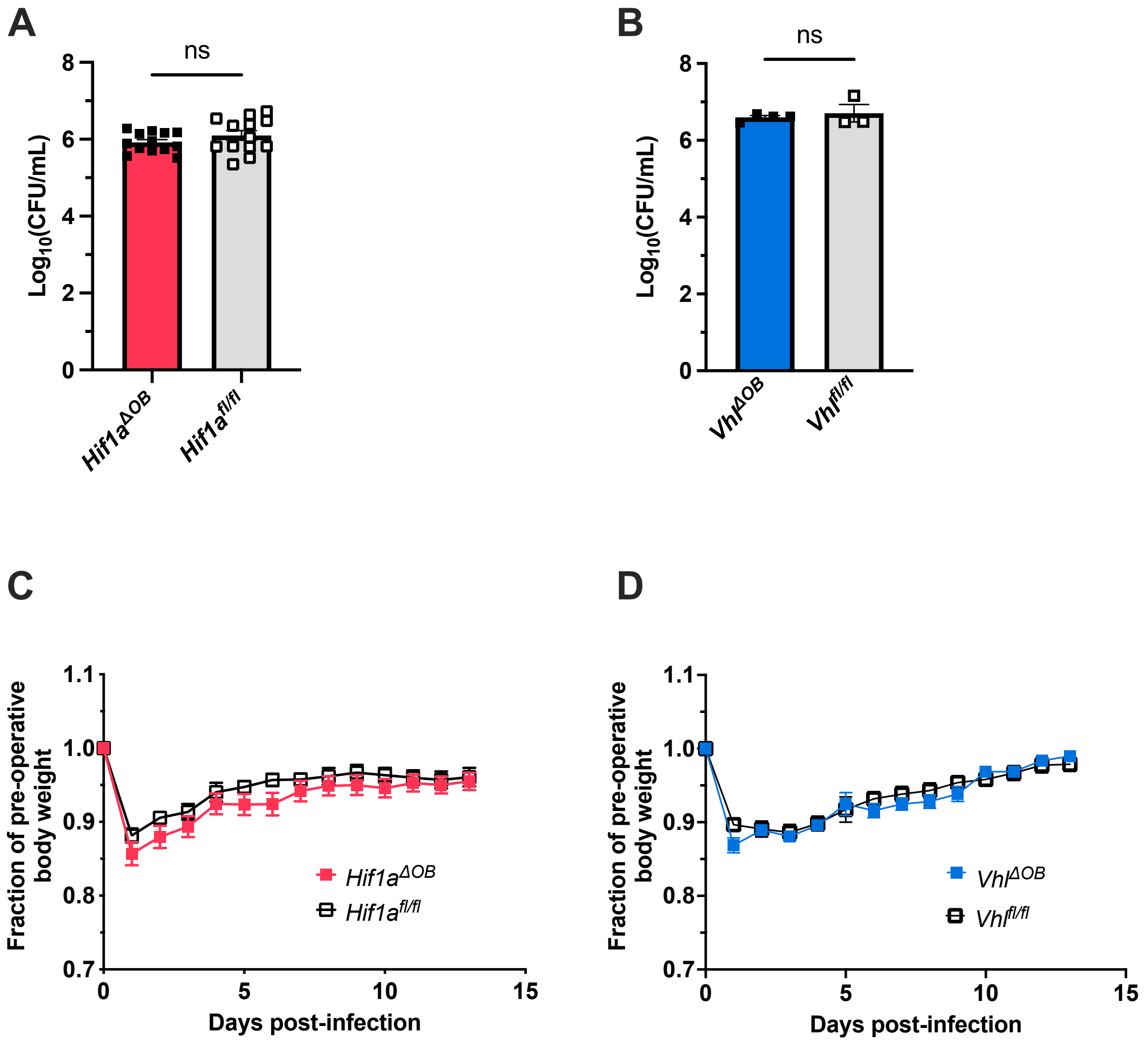

Figure S1: Conditional knockout of *Hif1a* or *Vhl* in the osteoblast lineage in male mice does not impact bacterial burdens at post-infection Day 14

Bacterial burdens in infected femurs of male mice containing conditional knockouts of *Hif1a* (**A**, *n*=13) and *Vhl* (**B**, *n*=3-4) were measured at post-infection Day 14. The colony forming units (CFU) were compared to those of Cre-negative littermate controls that were homozygous for the respective floxed alleles. ns denotes not significant ($p > 0.05$) as determined by unpaired, two-tailed Student's *t*-tests. The weights of the corresponding infected animals analyzed in (**A**) are

shown in (**C**) (*n*=13). The weights shown in (**D**) represent those of animals analyzed for changes in bone architecture (**Figure 2E**), as well as those analyzed for bacterial burdens in (**B**) (*n*=9-10). The weights were measured daily and normalized to the pre-operative, uninfected animal weights before comparison by two-way analysis of variance (ANOVA). The relative weights of mice with conditional knockout of *Hif1a* (**C**) and *Vhl* (**D**) were not significantly different from those of littermate controls. Error bars represent mean ± SEM.

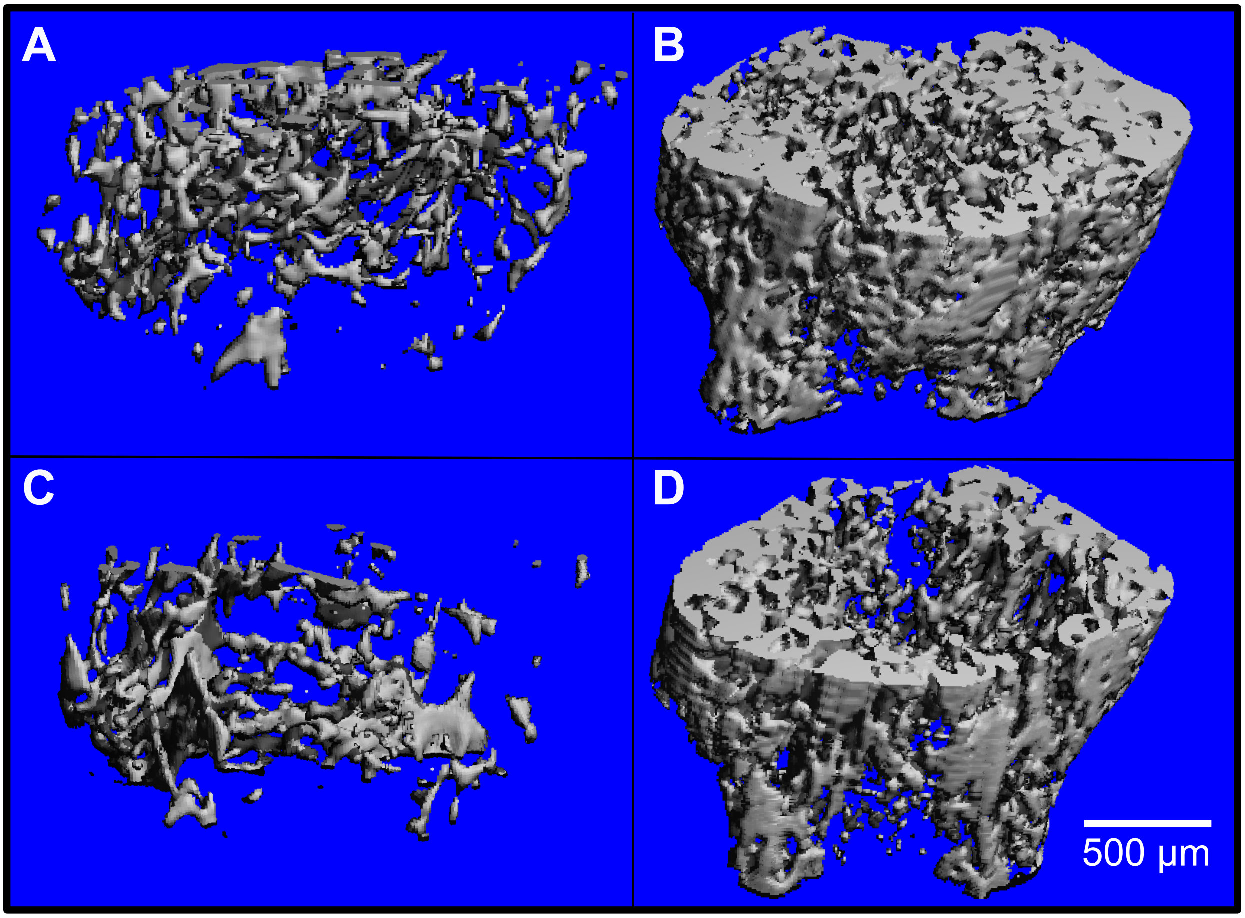

Figure 2: The duration of doxycycline withdrawal impacts trabecular bone volume in *Vhl*$^{\Delta OB}$ mice

MicroCT reconstructions of trabecular bone from the distal metaphysis of the right femur of a *Vhl*$^{fl/fl}$ control (**A**) is shown in relation to that of its corresponding *Vhl*$^{\Delta OB}$ littermate (**B**). The animals in (**A**) and (**B**) were both maintained on doxycycline until postnatal age 1 week, at which time doxycycline was no longer administered to allow for activation of the Cre recombinase. (**C**) and (**D**) represent equivalent regions of the trabecular bone of femurs from *Vhl*$^{fl/fl}$ and *Vhl*$^{\Delta OB}$ littermates for which doxycycline was withdrawn at postnatal age 4 weeks. All femurs analyzed are the right (contralateral) femur of mice at post-infection day 14.

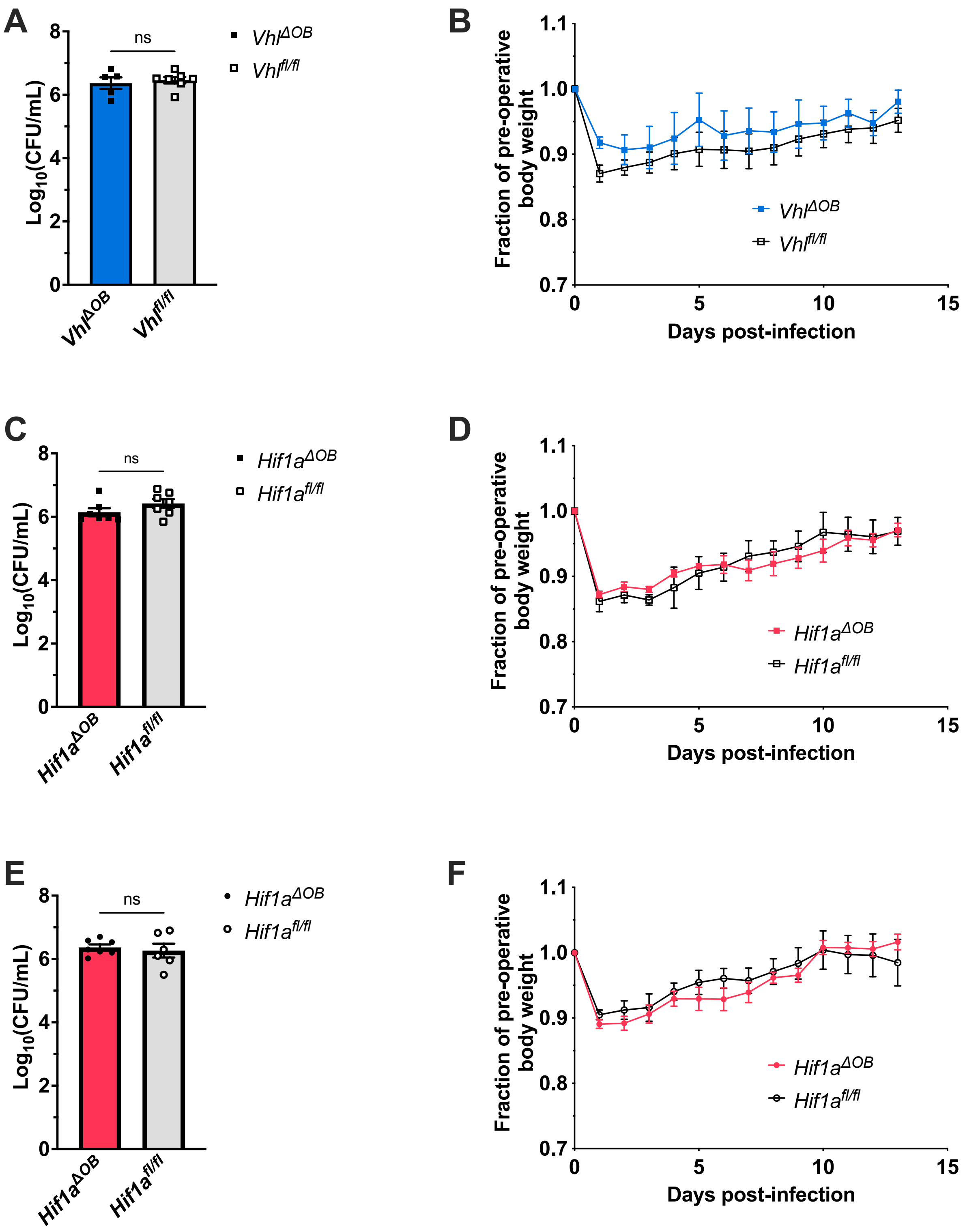

Figure S3: Bacterial burdens in mice with osteoblast-lineage conditional knockout of *Hif1a* or *Vhl* do not differ from controls with extended doxycycline withdrawal

Following withdrawal of doxycycline at postnatal age 1 week, bacterial burdens of male *Vhl*$^{\Delta OB}$ mice and *Vhl*$^{fl/fl}$ controls (**A**, *n*=5-7), male *Hif1a*$^{\Delta OB}$ mice and *Hif1a*$^{fl/fl}$ controls (**C**, *n*=7), and female *Hif1a*$^{\Delta OB}$ mice and *Hif1a*$^{fl/fl}$ controls (**E**, *n*=6,7) were measured in infected femurs at post-infection Day 14. Error bars represent mean ± SEM, and ns denotes not significant ($p > 0.05$) as determined by unpaired, two-tailed Student's *t*-tests with comparison to burdens of Cre-negative littermate controls. For each experiment in (**A**), (**C**), and (**E**), corresponding post-infection weights were recorded as a fraction of the uninfected, pre-operative weight (**B**, male *Vhl*$^{\Delta OB}$ mice and *Vhl*$^{fl/fl}$ controls; **D**, male *Hif1a*$^{\Delta OB}$ mice and *Hif1a*$^{fl/fl}$ controls; and **F**, female *Hif1a*$^{\Delta OB}$ mice and *Hif1a*$^{fl/fl}$ controls). For each assessment of weights over the course of the 14-day infection, no significant differences were observed between the conditional knockout animals and the corresponding control group as determined by two-way analysis of variance (ANOVA). Error bars represent mean ± SEM. CFU=colony forming units.

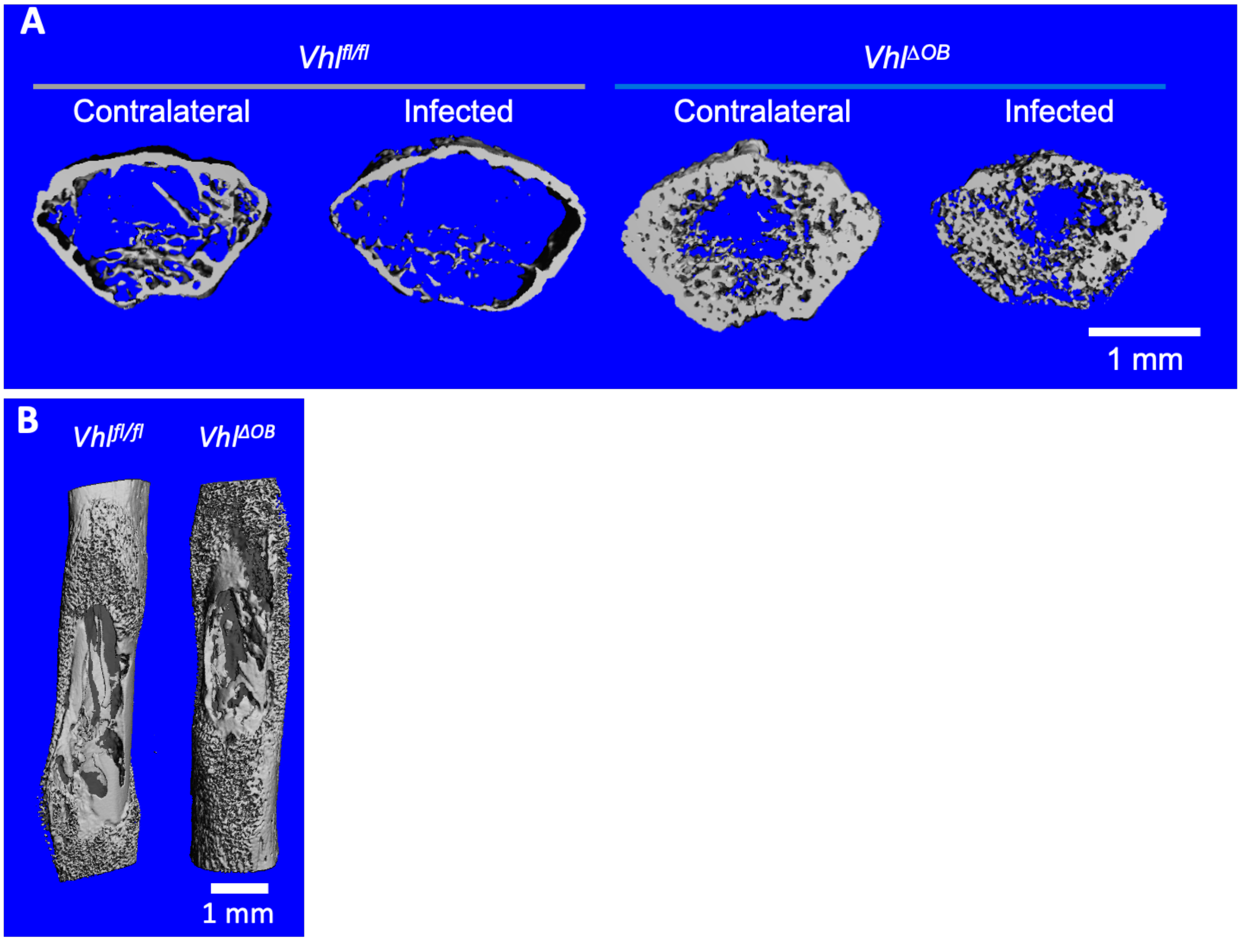

Figure S4: Representative microCT reconstructions from bones of *Vhl*$^{\Delta OB}$ mice and Cre-negative littermate control mice

(**A**) Representative microCT cross-sectional reconstructions through the distal metaphyses of infected and contralateral femurs of *Vhl*$^{\Delta OB}$ mice and Cre-negative littermate controls (*Vhl*$^{fl/fl}$ mice). A view is shown in cross-section to the long axis of the femur through the highly trabecularized region of the distal metaphysis and includes the cortical bone. The three-dimensional reconstruction represents 50 slices (approximately 0.05 mm thick) in the center of the 101 slices analyzed in the trabecular bone analysis. Both *Vhl*$^{\Delta OB}$ mice and *Vhl*$^{fl/fl}$ mice show cortical shell thinning, while the trabecular bone internal to this outer shell represents the sample analyzed by BV/TV in Figure 2B. (**B**) Representative microCT reconstructions of infected

femurs from *Vhl*$^{\Delta OB}$ mice with images from Cre-negative littermate controls (*Vhl*$^{fl/fl}$ mice). Bone segments show the anterolateral surface of the femur along the diaphysis of the bone from the proximal (top of image) metaphysis to the distal (bottom of image) physis, and the images primarily show the cortical shell and reactive bone formation.

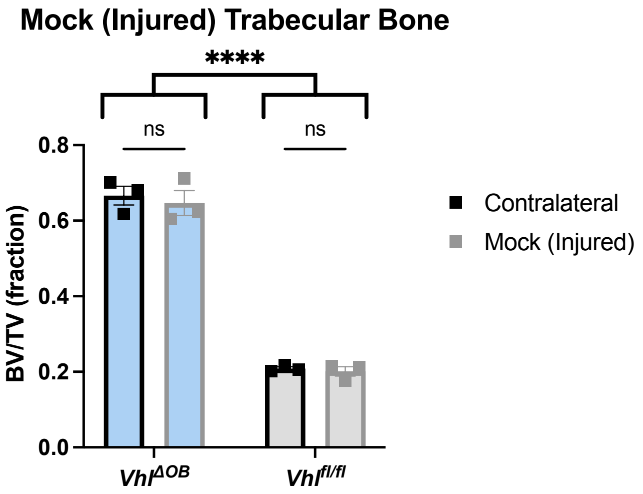

Figure S5: Trabecular bone loss does not occur in *Vhl*$^{\Delta OB}$ mice or Cre-negative littermate controls following mock infection

Trabecular bone volume per total volume (BV/TV) was assessed in the distal metaphysis of left femurs of *Vhl*$^{\Delta OB}$ and *Vhl*$^{fl/fl}$ littermates (*n*=3, male) following sterile mock infection and compared to the BV/TV of the trabecular bone in the contralateral femur. ****$p < 0.0001$ (comparison of bracketed genotypes as a whole without multiple comparisons) and ns denotes not significant ($p > 0$ .05) as determined by two-way analysis of variance (ANOVA). Error bars represent mean ± SEM.

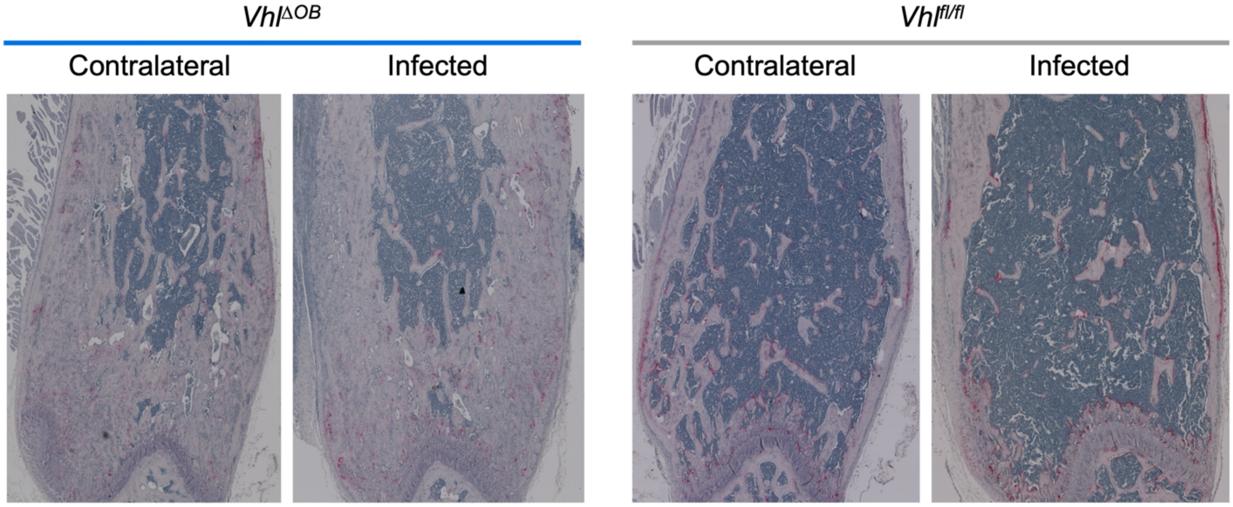

Figure S6: Representative images of TRAP-stained trabecular bone in infected and contralateral femurs of *Vhl*$^{\Delta OB}$ mice and *Vhl*$^{fl/fl}$ littermate control mice

Representative images of TRAP-stained trabecular bone in infected and contralateral femurs of *Vhl*$^{\Delta OB}$ mice and *Vhl*$^{fl/fl}$ littermate control mice are shown. Images to the left are those of *Vhl*$^{\Delta OB}$ mice with high volumes of trabecular bone. Images to the right are those of *Vhl*$^{fl/fl}$ littermate control mice. For both genotype sets shown, the left image is of the contralateral limb, and the right image is of the infected limb from the same animal.

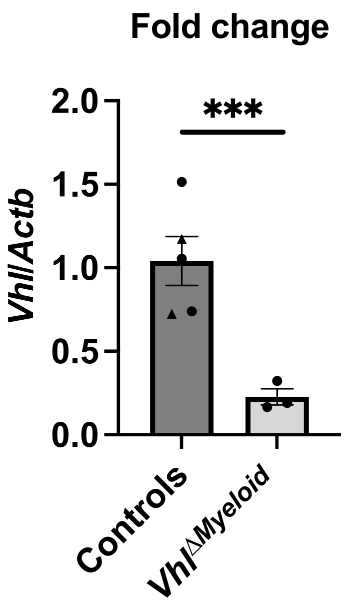

Figure S7: *Vhl* transcription is decreased in *Vhl*$^{\Delta Myeloid}$ mice compared to controls.

RT-PCR measurement of *Vhl* transcript levels in bone marrow-derived macrophages from *Vhl*$^{\Delta Myeloid}$ mice (*n*=3 mice) and controls (a combination of *Vhl*$^{fl/fl}$ littermates [*n*=2, triangles] and age-matched *LysMCre*$^{+/-}$mice [*n*=3, circles]). Transcripts are shown relative to the control gene *Actb* as determined by the $2^{-\Delta\Delta CT}$ method. ***$p < 0.001$ as determined by Student's *t*-test. Error bars represent mean ± SEM.

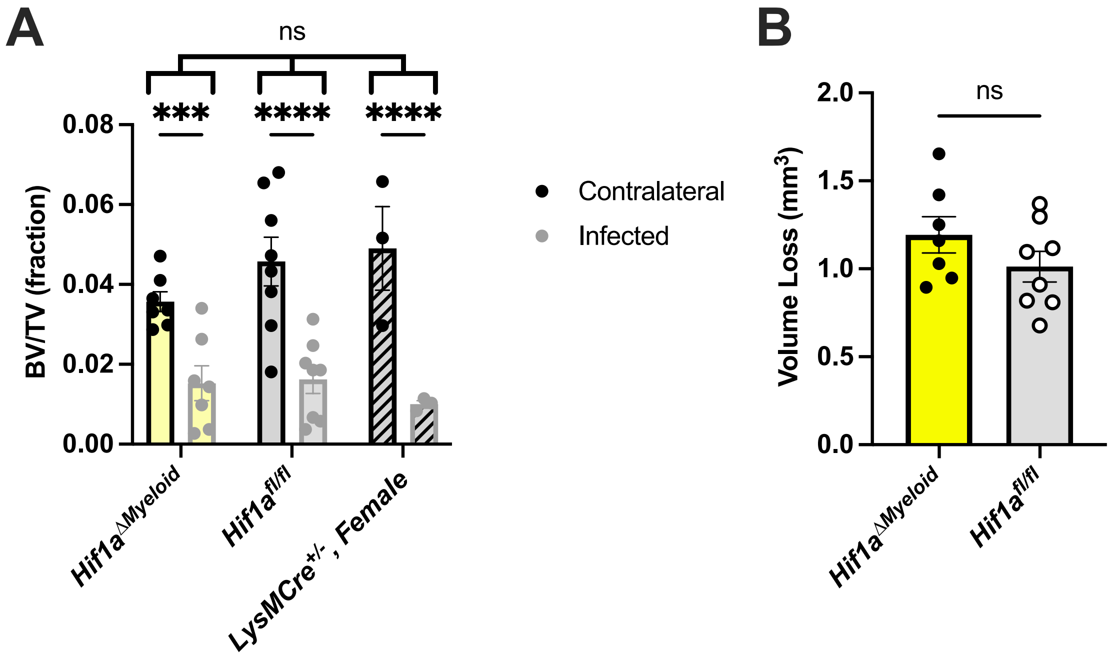

Figure S8: Trabecular bone changes in female *Hif1a*$^{\Delta Myeloid}$ mice do not differ from those of control mice

(**A**) The bone volume-per-total volume (BV/TV) in *Hif1a*$^{\Delta Myeloid}$, *Hif1a*$^{fl/fl}$ littermate control mice, and age-matched control mice (*n*=3-8, female) at post-infection Day 14 was measured in the trabecular bone of the distal metaphysis of infected and contralateral femurs. Infection significantly reduced BV/TV compared to contralateral femurs within all genotypes as compared by two-way analysis of variance (ANOVA) with correction for multiple comparisons. Moreover, genotypes (second variable of two-way ANOVA, denoted as 3 bracketed groups) did not significantly differ from one another. (**B**) For the littermate mice of (**A,** *Hif1a*$^{\Delta Myeloid}$ and littermate *Hif1a*$^{fl/fl}$ control mice, *n*=7-8) cortical bone destruction was measured at post-infection Day 14. ***$p < 0.001$, ****$p < 0.0001$, and ns denotes not significant ($p > 0.05$) as determined by two-way ANOVA with correction for multiple comparisons (**A**) or unpaired, two-tailed Student's *t*-test (**B**). Error bars represent mean ± SEM.

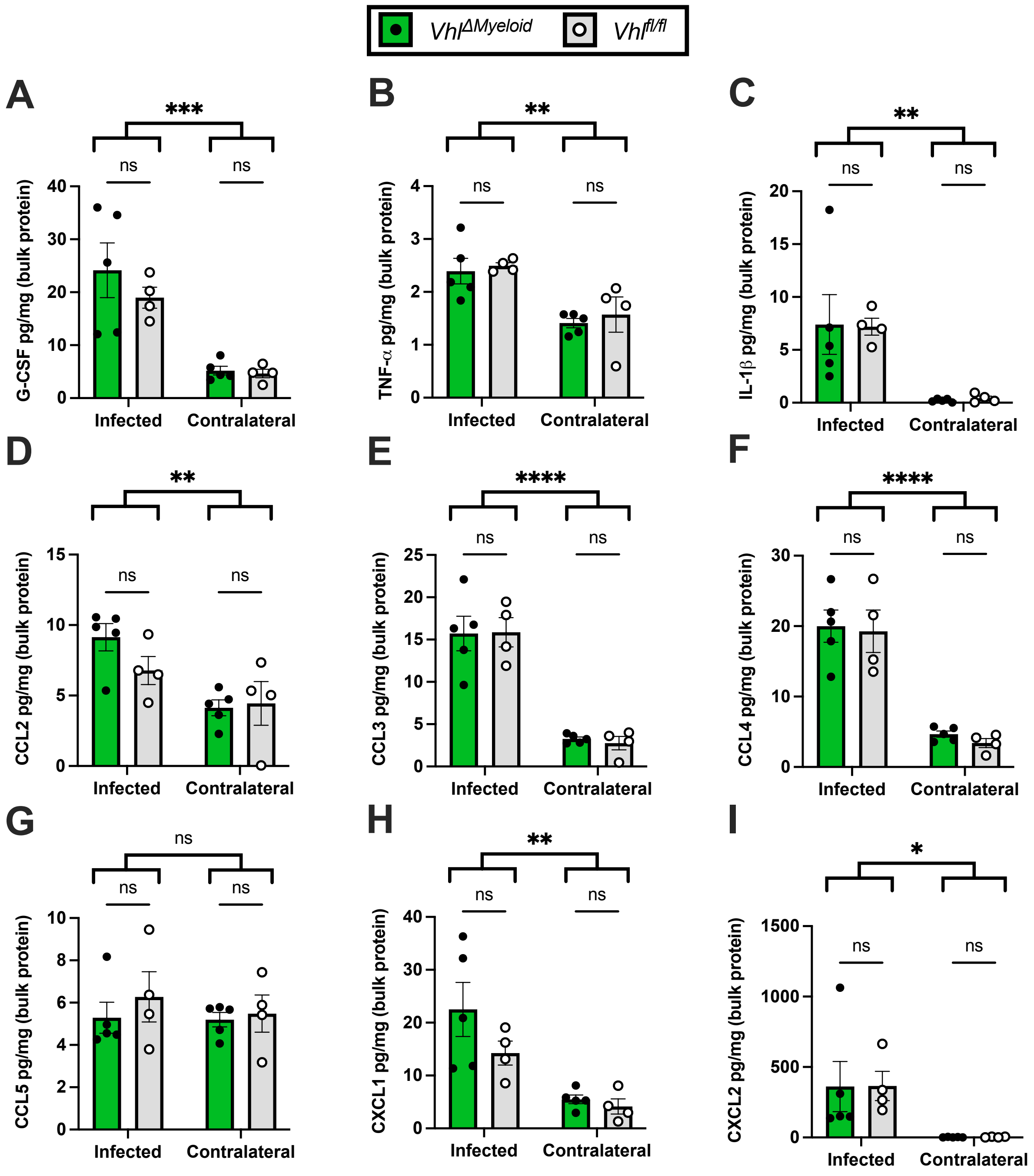

Figure S9: Conditional knockout of *Vhl* in myeloid cells does not alter cytokine abundance in infected femurs

Cytokine abundances in infected and contralateral femurs of *Vhl*$^{\Delta Myeloid}$ mice and Cre-negative littermate *Vhl*$^{fl/fl}$ control mice (*n*=4-5, female) at post-infection day 5 were measured using Luminex multiplexed cytokine analysis for G-CSF (**A**), TNF-α (**B**), IL-1β (**C**), CCL2 (**D**), CCL3

(**E**), CCL4 (**F**), CCL5 (**G**), CXCL1 (**H**), and CXCL2 (**I**). The differences between the groups were compared by two-way analysis of variance (ANOVA), which revealed that the laterality between infected and contralateral femurs (two bracketed groups) significantly differ in all but CCL5, as shown. Differences between genotypes were not observed by two-way ANOVA. *, $p < 0.05$, **$p < 0.01$, ***$p < 0.001$, ****$p < 0.0001$, and ns denotes difference did not meet the $p < 0.05$ standard for significance as determined by two-way ANOVA. Error bars represent mean ± SEM.
